# CLEMENT: genomic decomposition and reconstruction of non-tumor subclones

**DOI:** 10.1093/nar/gkae527

**Published:** 2024-06-26

**Authors:** Young-soo Chung, Seungseok Kang, Jisu Kim, Sangbo Lee, Sangwoo Kim

**Affiliations:** Department of Biomedical Systems Informatics, Brain Korea 21 PLUS Project for Medical Science, Yonsei University College of Medicine, Seoul 03722, Republic of Korea; Department of Biomedical Systems Informatics, Brain Korea 21 PLUS Project for Medical Science, Yonsei University College of Medicine, Seoul 03722, Republic of Korea; DataShape team, Inria Saclay Île-De-France, Palaiseau 91120, France; Department of Statistics, Seoul National University, Seoul 08826, Republic of Korea; Department of Biomedical Systems Informatics, Brain Korea 21 PLUS Project for Medical Science, Yonsei University College of Medicine, Seoul 03722, Republic of Korea; Department of Biomedical Systems Informatics, Brain Korea 21 PLUS Project for Medical Science, Yonsei University College of Medicine, Seoul 03722, Republic of Korea

## Abstract

Genome-level clonal decomposition of a single specimen has been widely studied; however, it is mostly limited to cancer research. In this study, we developed a new algorithm CLEMENT, which conducts accurate decomposition and reconstruction of multiple subclones in genome sequencing of non-tumor (normal) samples. CLEMENT employs the Expectation-Maximization (EM) algorithm with optimization strategies specific to non-tumor subclones, including false variant call identification, non-disparate clone fuzzy clustering, and clonal allele fraction confinement. In the simulation and *in vitro* cell line mixture data, CLEMENT outperformed current cancer decomposition algorithms in estimating the number of clones (root-mean-square-error = 0.58–0.78 versus 1.43–3.34) and in the variant-clone membership agreement (∼85.5% versus 70.1–76.7%). Additional testing on human multi-clonal normal tissue sequencing confirmed the accurate identification of subclones that originated from different cell types. Clone-level analysis, including mutational burden and signatures, provided a new understanding of normal-tissue composition. We expect that CLEMENT will serve as a crucial tool in the currently emerging field of non-tumor genome analysis.

## Graphical Abstract

**Graphical Abstract:**
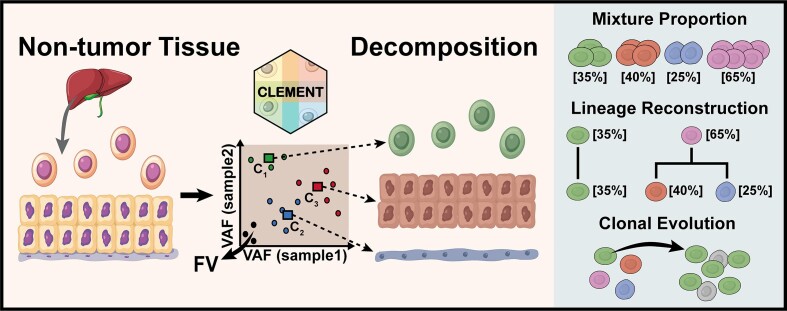

## Introduction

Poly-clonality within a single specimen and its accurate decomposition have been important concerns in genomic analysis. Most research efforts to address this issue have focused on cancer, in which multiple subclones give rise to genetically distinct populations of a single tumor, resulting in intratumoral heterogeneity (ITH) that is responsible for drug resistance, tumor relapse, and poor clinical outcomes (1). Several methods, such as PyClone (2), SciClone (3), PyClone-VI (4) and QuantumClone (5), have been developed for the accurate decomposition and reconstruction of the cancer subclones. Although different statistical models and optimization strategies have been employed, the conceptual assumption is largely limited to the use of clonally expanded somatic mutations, which are clearly identifiable in conventional genome sequencing.

Recent advances in genomic analysis of non-tumor (i.e. normal) tissues pose a new challenge in genomic decomposition. Accurate clonal decomposition in normal tissue is necessary as it provides an understanding of the molecular-level landscape of the developmental process (6) or patterns of mosaicism (7). Additionally, clone-level analysis is applicable to various of non-cancer disease, such as early developmental disorders or borderline premalignancies (8,9). While both tumor and non-tumor tissues in a single specimen have genetically distinct populations, fundamental differences in genomic characteristics and variant detectability lead to suboptimal results when applying existing methods to non-tumor decomposition. First, detection of somatic mutations in normal tissues is fraught with the low variant allele frequency (VAF) (<1–5%) (10), which causes numerous false calls. A series of brain mosaicism studies reported false positives ranging from 9.9 to 32.9% of total variants (11,12). As clone-specific mutations are the key evidence for decomposition, the erroneous variants should be considered and properly handled. Second, the genomic similarity among clones is higher in normal tissues due to the lower mutation rate and limited observable clone-specific mutations, making a deterministic assignment of clones difficult and negatively affects the estimation of clone numbers. Lastly, the absence of copy number alterations (CNA), important evidence of tumor decomposition, limits the information for clone identification and makes the entire algorithm rely solely on SNVs in normal tissues. Additionally, the lack of CNA alleviates the model complexity in relating VAF to cellular prevalence and warrants more efficient decomposition. These differences emphasize the need for a specialized method for the genomic decomposition of non-tumor samples.

In this study, we present a new method CLEMENT (CLonal decomposition using Expectation-Maximization algorithm Established in Non-Tumor diploid samples), for accurate decomposition and reconstruction of subclones in non-tumor tissues. We employed the following three core strategies to resolve the aforementioned problems: (1) measuring and parameterizing false positivity in the input variants to reduce noise in clone identification, (2) using fuzzy clustering to enable more flexible discrimination of genetically similar clones, and (3) setting restrictions on clonal fractions in the determination of clonal compositions (i.e. total clone fraction = 1, see Materials and methods for details) due to the absence CNAs. We observed the improved accuracy of CLEMENT in three independent, high-quality datasets: *in silico* simulations, *in vitro* cell-line mixture (13), and human datasets derived from multiple normal tissues using laser capture microdissection (LCM) (14). We anticipate that CLEMENT will provide a deeper understanding of genomic and tissue-level heterogeneity, mosaicisms, and the functional relatedness of somatic mutations in normal tissues.

## Materials and methods

### Overview of the CLEMENT algorithm

**Figure 1.**
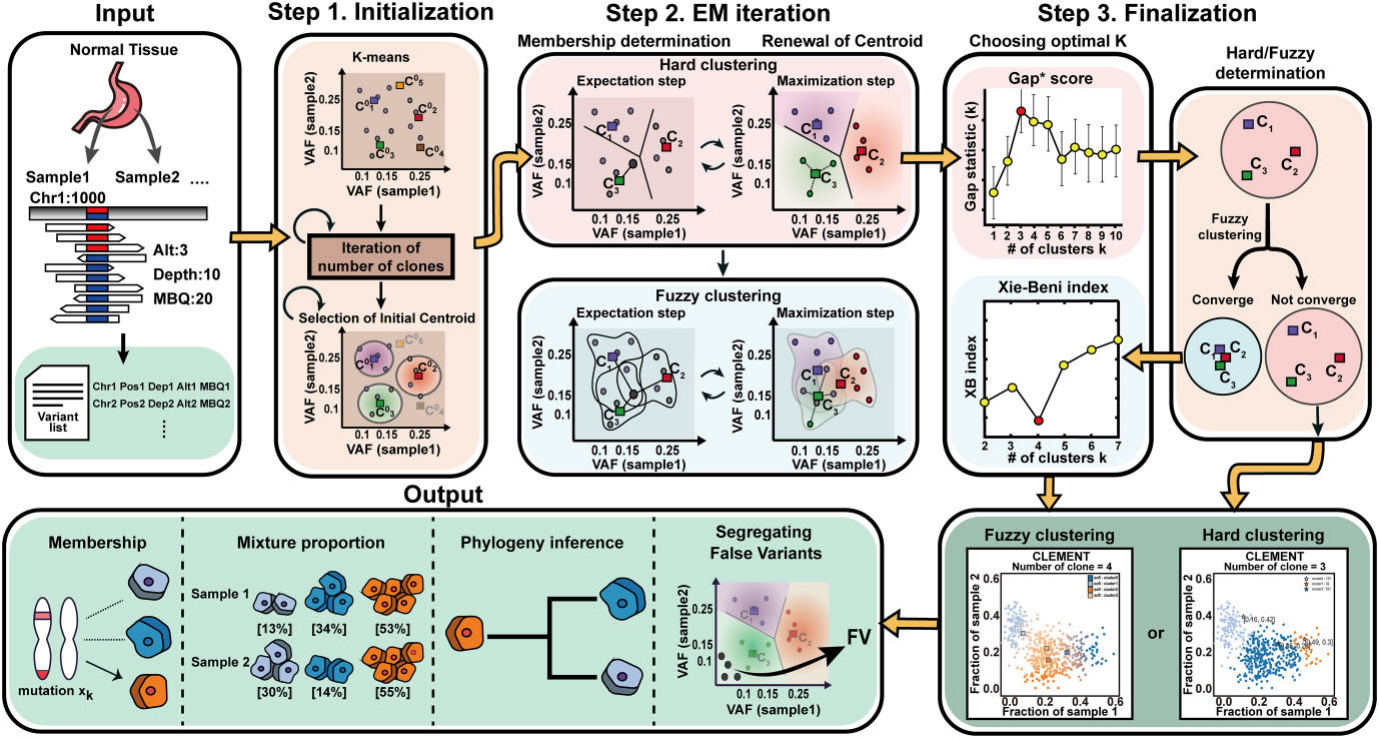
Overview of the CLEMENT workflow and core algorithms. Three steps (initialization, EM iteration and finalization) are depicted with defined input (variant information including total-, alternate read depth and base quality) and output (clone numbers, compositions, and variant membership). VAF: variant allele frequency.

CLEMENT consists of three major steps, as follows: (i) the initialization step that determines the initial number of clones using K-means clustering, (ii) the iteration step that searches for the optimal compositions of clones based on the Expectation-Maximization (EM) algorithm in a given number of clones and (iii) the finalization step to determine the optimal number of individual and ancestral clones (undifferentiated clones that harbor two or more individual clones) and their hierarchical clone structures (Figure 1). CLEMENT uses a list (or lists, if two or more samples are provided) of established somatic variants and their total read counts and alternate read counts as input, and outputs a list of individual and ancestral clones, and the membership of somatic variants with a visual representation. Detailed methods are formulated in the following sections.

#### Definitions of subclone and superclone

The term ‘clone’ refers to a set of cells that harbor unique characteristics in terms of mutation (5). In CLEMENT, we used variants as a genomic feature to define clones. Among the clones, we defined ‘*subclones*’ or *individual* clones, as clones that are genetically mutually exclusive (Supplementary Figure S1). The total cellular prevalence of subclones is 1.0 by definition. With the lack of CNAs and homozygosity of somatic mutations in normal tissue, the sum of VAFs is 0.5. In contrast, *ancestral* clones, or ‘*superclones’*, possess a clonal mutation that has been dispersed among their own subclones, where the proportions of clonal mutations are the sum of the proportions of subclones (15).

#### Basic mathematical definitions

Let $S = \{ {{{s}_1}, \ldots ,{{s}_m}} \}$ and $V = \{ {{{v}_1}, \ldots ,{{v}_n}} \}$ be the set of $m$ samples and $n$ somatic variants given to CLEMENT, respectively. User input


\begin{eqnarray*}{{{\boldsymbol{N}}}^{total}} = \left\{ {n_{i,j}^{total}:n_{i,j}^{total} = \ total\ read\ count\left( {{{s}_i},{{v}_j}} \right),\ 1 \le i \le m,\ 1 \le j \le n} \right\}\end{eqnarray*}

\begin{eqnarray*}{{{\boldsymbol{N}}}^{alt}} = \left\{ {n_{i,j}^{alt}:n_{i,j}^{alt}\ = \ alternate\ allele\ count\left( {{{s}_i},{{v}_j}} \right),\ 1 \le i \le m,\ 1 \le j \le n} \right\}\end{eqnarray*}

are multisets of the total read count (i.e. read depth) and alternate allele count of each sample and genomic position of variants. $n_{i,j}^{total}$ is doubled when ${{s}_i}$ is male and ${{v}_j}$ located at sex chromosome, to calibrate from the haploid to diploid data.

From them, we define ${\boldsymbol{F}} = \{ {{{f}_{i,j}}:{{f}_{i,j}} = {\mathrm{VAF}}( {{{s}_i},\ {{v}_j}} ) = \frac{{n_{i,j}^{alt}}}{{n_{i,j}^{total}}},1 \le i \le m,\ 1 \le j \le n,0 \le {{f}_{i,j}} \le 1} \}$ as a multiset of VAF values of the variant set $V$, allowing for duplication. During the algorithm, $k + 1$ clusters composed of $k$ true biologic clones and a cluster of false variant (FV) $C = \{ {{c}_1}, \ldots ,{{c}_k},{{c}_{FV}}\}$ are assumed, where each cluster occupies a subset of variants.

${{{\boldsymbol{\lambda }}}^i}$ and ${\boldsymbol{\Lambda }}$ are defined as a posterior probability matrix with elements denoted as ${{{\boldsymbol{\lambda }}}^i}( {{{{\mathrm{v}}}_{\mathrm{j}}},{{c}_y}} )$ and ${\boldsymbol{\Lambda }}( {{{{\mathrm{v}}}_{\mathrm{j}}},{{c}_y}} )$ ($1 \le j \le n,\ y = \{ {1, \ldots ,k,FV} \}$) with regard to single sample ${{s}_i}\ ( {1 \le i \le m} )$ and whole sample respectively, satisfying


\begin{eqnarray*}\mathop \sum \limits_{y = 1}^k {{{\boldsymbol{\lambda }}}^i}\left( {{{{\mathrm{v}}}_{\mathrm{j}}},{{c}_y}} \right) + \ {{{\boldsymbol{\lambda }}}^i}\left( {{{v}_j},{{c}_{FV}}} \right) = 1\end{eqnarray*}

and


\begin{eqnarray*}{\boldsymbol{\Lambda }}\left( {{{v}_j},{{c}_y}} \right) = \frac{{\mathop \prod \nolimits_{i = 1}^m {{{\boldsymbol{\lambda }}}^i}\left( {{{{\mathrm{v}}}_{\mathrm{j}}},{{c}_y}} \right)}}{{\mathop \sum \nolimits_{z = \left\{ {1, \ldots ,k,FV} \right\}} \mathop \prod \nolimits_{i = 1}^m {{{\boldsymbol{\lambda }}}^i}\left( {{{{\mathrm{v}}}_{\mathrm{j}}},{{c}_z}} \right)}}\end{eqnarray*}

Subsequently, we defined the membership function${\mathrm{\ }}{\boldsymbol{\Theta }} = V \times C \to \{ {\rho :0 \le \rho \le 1} \}$ by satisfying for each $v \in V$, $\mathop \sum \limits_{y = 1}^k {\boldsymbol{\Theta }}( {v,{{c}_y}} ) + {\boldsymbol{\Theta }}( {v,{{c}_{FV}}} ) = 1$, which is gathered from ${\boldsymbol{\Lambda }}$. For hard clustering, ${\boldsymbol{\Theta }}( {v,c} )$ is either $0$ or $1$; ${\boldsymbol{\Theta }}( {v,c} ) = 1$ if $v \in V$ is a member of $c \in C$, and ${\boldsymbol{\Theta }}( {v,c} ) = 0$ if $v\ \in \ V$ is not a member of $c\ \in \ C$ (see below). For fuzzy clustering, we set ${\boldsymbol{\Theta }} = \ {\boldsymbol{\Lambda }}$ to allow partial membership by assigning a posterior probability between 0 and 1 in E step (see below).

Lastly, let ${{{{\bf \mu }}}^{\mathrm{i}}}( {{{{\mathrm{c}}}_{\mathrm{y}}}} ){\mathrm{\ }}( {y = \{ {1, \ldots ,k,FV} \}} )$ be the centroid of a cluster ${{c}_y}$ in sample $i$, and ${\boldsymbol{{\rm M}}}( {{{{\mathrm{c}}}_{\mathrm{y}}}} ){\mathrm{\ }}( {y = \{ {1, \ldots ,k,FV} \}} )$ be the centroid in m-dimensional vector, comprised of $({{{{\bf \mu }}}^1}( {{{{\mathrm{c}}}_{\mathrm{y}}}} ), \ldots ,{{{{\bf \mu }}}^{\mathrm{m}}}( {{{{\mathrm{c}}}_{\mathrm{y}}}} )$), which is recalibrated in M step.

##### Step 1: Initialization

In the Initialization step, CLEMENT selects $k$ initial centroids, $k$ is iterated within range of [2, 10] (user adjustable), on the given data $S$$V$, *and*$F$, to provide a rough estimate of the clone structure for the next EM-iteration step.

First, CLEMENT performs the *K*-means clustering using the scikit-learn (version 1.0.2) package (16) to partition somatic variants ${}^\forall {v}_{j} \in V(1\ \le \ j\ \le \ n)$, an $m$-dimensional VAF vector $( {{{f}_{1,j}},\ \ldots ,{{f}_{m,j}}} )$, into $T$ (user adjustable; default = 10) clusters. Then, CLEMENT randomly selects $k\ ( {k \le T} )$ initial TP clusters out of $T$ given centroids, which implies a provisional clone set ${{C}^0} = \{ {c_1^0, \ldots ,c_k^0,c_{FV}^0} \}$. This random selection step is repeated for 10 times (user adjustable) for each $k$ to ensure extensive exploration.

##### Step 2: EM Iteration

This EM-iteration step takes the initial clone set ${{C}^0} = \{ {c_1^0, \ldots ,c_k^0,c_{FV}^0} \}$ from the aforementioned step, together with $S$, $V$, and $F$, and outputs a clone set ${{C}^{Max}} = \{ {c_1^{Max}, \ldots ,c_k^{Max},c_{FV}^{Max}} \}$ of the Maximum a Posteriori (MAP) probability. In this step, an alternative EM process is iterated 20 times (user adjustable) until it satisfies the stopping conditions (see the end of the section). The whole EM iteration step is conducted in two ways, as follows: first, in a hard clustering, and then in followed a fuzzy clustering (Figure 1, top middle). The final output of the EM iteration can be either from the initial hard clustering or the fuzzy clustering, which is determined later in Step 3 (Figure 1, bottom right).

##### E step

In the E step, CLEMENT assigns each data point to the $k$ clones, given the centroids of clusters as a latent variable. CLEMENT also considers the probability that a given variant is a sequencing artifact, or falsely called, aiming to exclude its assignment to true clones. To achieve this, we created an additional cluster with a fixed centroid (${{c}_{FV}}$) at the origin $(0, \ldots ,0$), consisting of false variants. This cluster is distinguishable from other clones $({{c}_1}, \ldots ,\ {{c}_k})$ because it does not represent a true biologic clone.

For $\forall i,\ j$, and $y = \{ {1, \ldots ,k,FV} \},$ posterior probability of data point belonging to clone $j$ in sample $i$ are calculated based on Bayesian theorem.


(1) \begin{eqnarray*}\begin{array}{*{20}{c}} {{\mathrm{\ }}P\left( {{{c}_y}{\mathrm{|}}{{v}_j}} \right) = {{\lambda }^i}\left( {{{v}_j},{{c}_y}} \right) = \ \frac{{P\left( {{{v}_j}{\mathrm{|}}{{c}_y}} \right)P\left( {{{c}_y}} \right)}}{{\mathop \sum \nolimits_z P\left( {{{v}_j}{\mathrm{|}}{{c}_z}} \right)P\left( {{{c}_z}} \right)}}} \end{array}\end{eqnarray*}

where $P( {{{v}_j}{\mathrm{|}}{{c}_y}} )$, $P( {{{c}_y}} )$ denotes likelihood and prior probability, respectively.

Meanwhile, a variant ${{v}_j}$ in sample $i$ can be categorized as true positive (TP), false positive (FP), true negative (TN) or false negative (FN).

If ${\mathrm{n}}_{{\mathrm{i}},{\mathrm{j}}}^{{\mathrm{alt}}} \ne 0$, ${{v}_j}$ is regarded either TP or FP. For subclone ${{c}_y}\ ( {y = \{ {1, \ldots ,k,FV} \}} )$ that satisfies ${{{\boldsymbol{\mu }}}^i}( {{{c}_y}} ) \ne 0$ in sample $i$ (TP), the likelihood of ${{v}_j}$ in subclone ${{c}_y}$ ($ = P({{v}_j}|{{c}_y})$) follows beta-binomial distribution, represented as ${{{{\bf L}}}_{{{\bf BetaBin}}}}( {n_{i,j}^{alt}{\mathrm{|}}n_{i,j}^{total},{{{\mathrm{\alpha }}}_{{\mathrm{i}},{\mathrm{y}}}},{{{\mathrm{\beta }}}_{{\mathrm{i}},{\mathrm{y}}}}} ){\mathrm{\ }} = P( {X = n_{i,j}^{alt}} ),\ X \sim {{\bf BetaBin}}( {n_{i,j}^{total},{{{\mathrm{\alpha }}}_{{\mathrm{i}},{\mathrm{y}}}},{{{\mathrm{\beta }}}_{{\mathrm{i}},{\mathrm{y}}}}} )$, where parameters are set as ${{{\mathrm{\alpha }}}_{{\mathrm{i}},{\mathrm{y}}}} = {{{{\bf \mu }}}^{\mathrm{i}}}( {{{{\mathrm{c}}}_{\mathrm{y}}}} ) \cdot {\mathrm{n}}_{{\mathrm{i}},{\mathrm{j}}}^{{\mathrm{total}}}$ and ${{{\mathrm{\beta }}}_{{\mathrm{i}},{\mathrm{y}}}} = (1 - {{{{\bf \mu }}}^{\mathrm{i}}}( {{{{\mathrm{c}}}_{\mathrm{y}}}} )) \cdot {\mathrm{n}}_{{\mathrm{i}},{\mathrm{j}}}^{{\mathrm{total}}}$ to maximize the likelihood if ${{v}_j}$ is located in the centroid. Because setting ${\mathrm{\hat{\alpha }}},{\mathrm{\ \hat{\beta }}}$ where 0 = $ {\frac{{\partial {{{{\bf L}}}_{{{\bf BetaBin}}}}}}{{\partial {\mathrm{\alpha }}}}} |{{{\mathrm{\ }}}_{{\mathrm{\hat{\alpha }}},\widehat {{\mathrm{\ \beta }}}}}$ and $0 = {\frac{{\partial {{{{\bf L}}}_{{{\bf BetaBin}}}}}}{{\partial {\mathrm{\beta }}}}} |{{{\mathrm{\ }}}_{{\mathrm{\hat{\alpha }}},\widehat {{\mathrm{\ \beta }}}}}$ requires a another computational load represented as Newton-Raphson method, we simply approximated to mean of the beta-binomial model coincides the mean of the observed data, ${{{{\bf \mu }}}^{\mathrm{i}}}( {{{{\mathrm{c}}}_{\mathrm{y}}}} )$ (17). Users can set the multiplication constant ${\boldsymbol{c}}$ to ${{{\mathrm{\alpha }}}_{{\mathrm{i}},{\mathrm{y}}}}$ and ${{{\mathrm{\beta }}}_{{\mathrm{i}},{\mathrm{y}}}}$ through the user input if input data is significantly condensed or dispersed. Meanwhile, for subclone ${{c}_y}\ ( {y = \{ {1, \ldots ,k,FV} \}} )$ that satisfies ${{{\boldsymbol{\mu }}}^i}( {{{c}_y}} ) = 0$ in sample $i$ (FP), the likelihood follows binomial distribution, ${{\bf B}}( {n_{i,j}^{total},\ {{p}_{SE}}} )$, where ${{p}_{SE}}$ stands for sequencing error probability of ${{v}_j}$ in sample $i$, inferred from base quality (BQ) score provided by the user input. If the users do not provide any input, it is set to 0.01 by default (18). So, likelihood of ${{v}_j}$ in cluster ${{c}_y}$ ($ = P({{v}_j}|{{c}_y})$) in sample $i$ is represented as ${{{{\bf L}}}_{{\bf B}}}( {n_{i,j}^{alt}{\mathrm{|}}n_{i,j}^{total},{{p}_{SE}}} ) = P( {X = n_{i,j}^{alt}} ),\ X \sim {{\bf B}}( {n_{i,j}^{total},{{p}_{SE}}} )$. Eq. 1 is rewritten in eq. 2–1 and eq. 2–2.

For ${{c}_y}$ that satisfies ${{{\boldsymbol{\mu }}}^i}( {{{c}_y}} ) \ne 0$ (TP),


(2-1) \begin{eqnarray*}{{{\boldsymbol{\lambda }}}^i}\left( {{{v}_j},{{c}_y}} \right) = \ \frac{{{{{{\bf L}}}_{{{\bf BetaBin}}}}\left( {{\mathrm{n}}_{{\mathrm{i}},{\mathrm{j}}}^{{\mathrm{alt}}}{\mathrm{|}}n_{ij}^{total},{{{\mathrm{\alpha }}}_{{\mathrm{i}},{\mathrm{y}}}},{\mathrm{\ }}{{{\mathrm{\beta }}}_{{\mathrm{i}},{\mathrm{y}}}}} \right) \cdot {\mathrm{P}}\left( {{{c}_y}} \right)}}{{\mathop \sum \nolimits_{{{{\boldsymbol{\mu }}}^i}\left( {{{c}_z}} \right) \ne 0} {{{{\bf L}}}_{{{\bf BetaBin}}}}\left( {{\mathrm{n}}_{{\mathrm{i}},{\mathrm{j}}}^{{\mathrm{alt}}}{\mathrm{|}}n_{i,j}^{total},{{{\mathrm{\alpha }}}_{{\mathrm{i}},{\mathrm{z}}}},{\mathrm{\ }}{{{\mathrm{\beta }}}_{{\mathrm{i}},{\mathrm{z}}}}} \right) \cdot {\mathrm{P}}\left( {{{c}_z}} \right) + \ \mathop \sum \nolimits_{{{{\boldsymbol{\mu }}}^i}\left( {{{c}_{z^{\prime}}}} \right) = 0} {{{{\bf L}}}_{{\bf B}}}\left( {n_{i,j}^{alt}{\mathrm{|}}n_{i,j}^{total},{{p}_{SE}}} \right){\mathrm{\ }} \cdot {\mathrm{P}}\left( {{{c}_{z^{\prime}}}} \right)}}\end{eqnarray*}

and for ${{c}_y}$ that satisfies ${{{\boldsymbol{\mu }}}^i}( {{{c}_y}} ) = 0$ (FP),


(2-2) \begin{eqnarray*}\ {{{\boldsymbol{\lambda }}}^i}\left( {{{v}_j},{{c}_y}} \right) = \ \frac{{{{{{\bf L}}}_{{\bf B}}}\left( {n_{i,j}^{alt}{\mathrm{|}}n_{i,j}^{total},{{p}_{SE}}} \right){\mathrm{\ }} \cdot {\mathrm{P}}\left( {{{c}_y}} \right)}}{{\mathop \sum \nolimits_{{{{\boldsymbol{\mu }}}^i}\left( {{{c}_z}} \right) \ne 0} {{{{\bf L}}}_{{{\bf BetaBin}}}}\left( {{\mathrm{n}}_{{\mathrm{i}},{\mathrm{j}}}^{{\mathrm{alt}}}{\mathrm{|}}n_{i,j}^{total},{{{\mathrm{\alpha }}}_{{\mathrm{i}},{\mathrm{z}}}},{\mathrm{\ }}{{{\mathrm{\beta }}}_{{\mathrm{i}},{\mathrm{z}}}}} \right) \cdot {\mathrm{P}}\left( {{{c}_z}} \right) + \ \mathop \sum \nolimits_{{{{\boldsymbol{\mu }}}^i}\left( {{{c}_{z^{\prime}}}} \right) = 0} {{{{\bf L}}}_{{\bf B}}}\left( {n_{i,j}^{alt}{\mathrm{|}}n_{i,j}^{total},{{p}_{SE}}} \right){\mathrm{\ }} \cdot {\mathrm{P}}\left( {{{c}_{z^{\prime}}}} \right)}}\end{eqnarray*}

The prior probability ${\mathrm{P}}( {{{c}_{z^{\prime}}}} )$ for each ${{c}_{z^{\prime}}}$ satisfying${\mathrm{\ }}{{{\boldsymbol{\mu }}}^i}( {{{c}_{z^{\prime}}}} ) = 0$ is set 0.01 by default (19), but the user can adjust this parameter by tuning the option. ${\mathrm{P}}( {{{c}_z}} )$ that satisfies${\mathrm{\ }}{{{\boldsymbol{\mu }}}^i}( {{{c}_z}} ) \ne 0$ is set to $\frac{{1 - \sum {\mathrm{P}}( {{{{\mathrm{c}}}_{{\mathrm{z^{\prime}}}}}} )}}{{{\mathrm{n}}( {\mathrm{z}} )}}$ to satisfy the sum of prior to be 1.

If ${\mathrm{n}}_{{\mathrm{i}},{\mathrm{j}}}^{{\mathrm{alt}}} = 0$, ${{v}_j}$ is either FN or TN for sample $i$. In case of FN, likelihood of ${{v}_j}$ in subclone ${{c}_y}$ ($ = P({{v}_j}|{{c}_y})$) in sample $i$ is ${{{{\bf L}}}_{{{\bf BetaBin}}}}( {0{\mathrm{|}}n_{i,j}^{total},{{{\mathrm{\alpha }}}_{{\mathrm{i}},{\mathrm{y}}}},{\mathrm{\ }}{{{\mathrm{\beta }}}_{{\mathrm{i}},{\mathrm{y}}}}} )$ of beta-binomial distribution as forementioned. Likewise, likelihood of ${{v}_j}$ being TN is derived from binomial distribution mentioned in FP, calculated as ${{{{\bf L}}}_{{\bf B}}}( {0{\mathrm{|}}n_{i,j}^{total},{{p}_{SE}}} )$ .

For ${{c}_y}$ that satisfies$\ {{{\boldsymbol{\mu }}}^i}( {{{c}_y}} ) \ne 0$ (FN),


(2-3) \begin{eqnarray*}{{{\boldsymbol{\lambda }}}^i}\left( {{{v}_j},{{c}_y}} \right) = \ \frac{{{{{{\bf L}}}_{{{\bf BetaBin}}}}\left( {0{\mathrm{|}}n_{i,j}^{total},{{{\mathrm{\alpha }}}_{{\mathrm{i}},{\mathrm{y}}}},{\mathrm{\ }}{{{\mathrm{\beta }}}_{{\mathrm{i}},{\mathrm{y}}}}} \right) \cdot {\mathrm{P}}\left( {{{c}_y}} \right)}}{{\mathop \sum \nolimits_{{{{\boldsymbol{\mu }}}^i}\left( {{{c}_z}} \right) \ne 0} {{{{\bf L}}}_{{{\bf BetaBin}}}}\left( {0{\mathrm{|}}n_{i,j}^{total},{{{\mathrm{\alpha }}}_{{\mathrm{i}},{\mathrm{z}}}},{\mathrm{\ }}{{{\mathrm{\beta }}}_{{\mathrm{i}},{\mathrm{z}}}}} \right) \cdot {\mathrm{P}}\left( {{{c}_z}} \right) + \mathop \sum \nolimits_{{{{\boldsymbol{\mu }}}^i}\left( {{{c}_{z^{\prime}}}} \right) = 0} {{{{\bf L}}}_{{\bf B}}}\left( {0{\mathrm{|}}n_{i,j}^{total},{{p}_{SE}}} \right) \cdot {\mathrm{P}}\left( {{{c}_{z^{\prime}}}} \right)\ }}\end{eqnarray*}

and for ${{c}_y}$ that satisfies ${{{\boldsymbol{\mu }}}^i}( {{{c}_y}} ) = 0$ (TN),


(2-4) \begin{eqnarray*}{{{\boldsymbol{\lambda }}}^i}\left( {{{v}_j},{{c}_y}} \right) = \ \frac{{{{{{\bf L}}}_{{\bf B}}}\left( {0{\mathrm{|}}n_{i,j}^{total},{{p}_{SE}}} \right) \cdot {\mathrm{P}}\left( {{{c}_y}} \right)}}{{\mathop \sum \nolimits_{{{{\boldsymbol{\mu }}}^i}\left( {{{c}_z}} \right) \ne 0} {{{{\bf L}}}_{{{\bf BetaBin}}}}\left( {0{\mathrm{|}}n_{i,j}^{total},{{{\mathrm{\alpha }}}_{{\mathrm{i}},{\mathrm{z}}}},{\mathrm{\ }}{{{\mathrm{\beta }}}_{{\mathrm{i}},{\mathrm{z}}}}} \right) \cdot {\mathrm{P}}\left( {{{c}_z}} \right) + \mathop \sum \nolimits_{{{{\boldsymbol{\mu }}}^i}\left( {{{c}_{z^{\prime}}}} \right) = 0} {{{{\bf L}}}_{{\bf B}}}\left( {0{\mathrm{|}}n_{i,j}^{total},{{p}_{SE}}} \right) \cdot {\mathrm{P}}\left( {{{c}_{z^{\prime}}}} \right)\ }}\end{eqnarray*}

Regarding the prior probability, sum of ${\mathrm{P}}( {{{c}_{z^{\prime}}}} )$ where ${{{\boldsymbol{\mu }}}^i}( {{{c}_{z^{\prime}}}} ) = 0$ (TN) is basically set 0.99 with an identical value for each clone (user adjustable), and ${\mathrm{P}}( {{{c}_z}} )$ where ${{{\boldsymbol{\mu }}}^i}( {{{c}_z}} ) \ne 0$ (FN) is set to $\frac{{1 - \mathop \sum \nolimits_{{{\mu }^i}( {{{c}_{z^{\prime}}}} ) = 0} P( {{{c}_{z^{\prime}}}} )}}{{n( z )\ }}$ to reflect the real-world knowledge (19).

Finally, ${{v}_j}$ is assigned to clone ${{c}_{\hat{y}}}$.


(3) \begin{eqnarray*}\begin{array}{*{20}{c}} {\hat{y} = \mathop {argmax}\limits_y \mathop \prod \limits_{i = 1}^m {{{\boldsymbol{\lambda }}}^i}\left( {{{v}_j},{{c}_y}} \right) = \ \mathop {argmax}\limits_y \ \Lambda \left( {{{v}_j},{{c}_y}} \right)} \end{array}\end{eqnarray*}

In hard clustering,


(4) \begin{eqnarray*}\begin{array}{@{}*{2}{c}@{}} {\Theta \left( {{{v}_j},{{c}_y}} \right) = \ \left[ {\begin{array}{@{}*{2}{c}@{}} 1&{,\ y = \hat{y}}\\ 0&{,\ y \ne \hat{y}} \end{array}} \right]\ for\ \forall j,y} \end{array}\end{eqnarray*}

In fuzzy clustering,


(5) \begin{eqnarray*}\begin{array}{*{20}{c}} {\Theta \left( {{{v}_j},{{c}_y}} \right) = \Lambda \left( {{{v}_j},{{c}_y}} \right)\ for\ \forall j,y} \end{array}\end{eqnarray*}

##### M step

In the M step, CLEMENT updates the centroid of each clone using the membership calculated in the E step as below:


(6) \begin{eqnarray*}\begin{array}{*{20}{c}} {{{{{\bf \mu }}}^{\mathrm{i}}}\left( {{{{\mathrm{c}}}_{\mathrm{y}}}} \right) = \frac{{\mathop \sum \nolimits_{{\mathrm{j}} = 1}^{\mathrm{n}} {{f}_{i,j}}{\boldsymbol{\Theta }}\left( {{{{\mathrm{v}}}_{\mathrm{j}}},{{{\mathrm{c}}}_{\mathrm{y}}}} \right){\mathrm{\ }}}}{{\mathop \sum \nolimits_{{\mathrm{j}} = 1}^{\mathrm{n}} {\boldsymbol{\Theta }}\left( {{{{\mathrm{v}}}_{\mathrm{j}}},{{{\mathrm{c}}}_{\mathrm{y}}}} \right)}}\ for\ \forall i,y} \end{array}\end{eqnarray*}

##### Distinguishing the individual clone and ancestral clone

After updating the centroids, CLEMENT classifies all the clones into either (i) an *individual clone*${{C}^{ind}}$ (${{C}^{ind}} \subseteq C$, $n({{C}^{ind}}) = k^{\prime}$), which is an independent clone that is separated from the other clones, or (ii) an *ancestral clone*${{C}^{anc}}$ (${{C}^{anc}} \subseteq C$, $n{\mathrm{\ }}({{C}^{anc}} \cap {{C}^{ind}}) = 0,$$n({{C}^{anc}}) = k - \ k^{\prime}$, Supplementary Figure S2), which is an undifferentiated clone that is composed of two or more individual clones. As mentioned earlier (see Basic mathematical definitions), CLEMENT chooses a set of clones from all possible combinations, the sum of whose centroids is 0.5. These set of clones are regarded as individual clones, or subclones (*subclone rule*, Eq. (7-1)).


(7-1) \begin{eqnarray*}\begin{array}{*{20}{c}} {\mathop \sum \limits_{y\ = \ 1}^{k^{\prime}} {{{{\bf \mu }}}^i}\left( {{{{\mathrm{c}}}_{\mathrm{y}}}} \right) = 0.5\ for\ {{{\mathrm{c}}}_{\mathrm{y}}} \in {{C}^{ind}},\ \forall i} \end{array}\end{eqnarray*}

CLEMENT employs multisample *t*-test with null hypothesis $\mathop \sum \limits_{y\ = \ 1}^{k^{\prime}} {{{{\bf \mu }}}^i}( {{{{\mathrm{c}}}_{\mathrm{y}}}} )$ – 0.5 = 0, with significance level = 0.01 and degree of freedom = $\mathop \sum \limits_{y = 1}^{k^{\prime}} n( {{{c}_y}} ) - k^{\prime}$. If variance of each cluster is not identical, an alternative degree of freedom is used (20). *t* values are derived from difference of group averages by dividing standard error of difference. If the statistics do not reject the null hypothesis, it ensures the sum of centroids is regarded as 0.5. When more than one combination of clusters satisfies the condition, CLEMENT selects the set with the highest *P* value on the multisample t-test, which supports the null hypothesis most strongly.

Ancestral clone ${{{\mathrm{c}}}_{\mathrm{z}}}$ is cluster of clonal mutations, whose proportion is sum of its subclones ${{{\mathrm{c}}}_{\mathrm{u}}}$. CLEMENT establishes superclone–subclone structure (phylogeny inference) by (eq. 7–2), in other words, sum rule (15).


(7-2) \begin{eqnarray*} M ({\mathrm{c}}_{z}) = \sum \limits_{{c}_{u} \in {C}^{\prime\,ind}} {M}(c_{u})\text{for}\ c_{z} \in {C}^{anc},\ C^{\prime\,ind} \subset \,{C}^{ind} \end{eqnarray*}

CLEMENT also employs another multisample *t*-test, assuming a null hypothesis that the mean value of the superclone is equal to the sum of mean values of the subclones, as previously described.

If any of (Eq. (7-1)) or (Eq. (7-2)) is unsatisfied, the iteration stops and restarts with Step 1 using another provisional clone set ${{C}^0} = \{ {c_1^0, \ldots ,c_k^0,c_{FV}^0} \}$. Otherwise, the E–M process continues until convergence.

##### Determination of convergence

The EM iteration stops if the following conditions are satisfied:

- A. The number of EM iterations is >10 times (user adjustable), AND:
- B-1. The gap between the Maximum a Posteriori probability of two successive steps is <1%, OR
- B-2. The gaps between all centroids of two successive steps are <0.01, OR
- B-3. The membership matrix ${\boldsymbol{\Theta }}$ retains same for two successive steps.

##### Step 3: Finalization

After two rounds of the EM iteration, CLEMENT determines whether the clustering results from the initial hard clustering or the secondary fuzzy clustering will be used as output (Figure 1, bottom right). Among the hard clustering results, CLEMENT uses Gap* Statistics (21) to choose optimal $k$, where the intra-cluster variation is minimized and inter-cluster variation is maximized. If Jaccard similarity between ${\boldsymbol{\Lambda }}( {v,{{c}_{{{y}_1}}}} )$ and ${\boldsymbol{\Lambda }}( {v,{{c}_{{{y}_2}}}} )$ exceeds 0.2 for $\exists {{y}_1},{{y}_2}\ ( {1\ \le \ {{y}_1},{{y}_2}\ \le \ k} )$, CLEMENT selects the result from fuzzy clustering; otherwise, the hard clustering results are retained. Finally, variants in ${{c}_{FV}}$ are designated as false variants (FV), in the other words, sequencing artifacts. The remaining variants are treated as true variants (TV).

### Test set preparation

#### Test set preparation

We used three independent test sets to measure the performance of CLEMENT and other tools, as follows: (i) a simulated dataset (SimData), (ii) an *in vitro* cell line mixture dataset (CellData) and (iii) real human multi-clone microdissected tissues (BioData).

#### Simulated dataset (SimData)

The SimData was made up of hypothetical clones and computationally introduced clone-specific somatic mutations thereof, based on two assumptions for non-tumor conditions: (a) absence of CNAs, and (b) absence of homozygote variants. Test sets were constructed with a random choice of (i) the number of clones ($k:2 \le k \le 7$) and (ii) the number of samples ($i:{\mathrm{\ }}1 \le i \le 3$) under the discrete uniform distribution. The number of clones and samples is limited arbitrarily for the economic computational burden of parallel benchmarking. The total number of variants and the mean read-depth for the benchmark were chosen as 500 and 125, to reflect the 125× DNA sequencing that is commonly used in real datasets.

Two types of datasets, SimData-decoy and SimData-lump, were generated computationally. First, for the SimData-decoy dataset, the proportional distribution of $k$ clones in sample ${\mathrm{i}}$ was randomly drawn from the Dirichlet distribution with shape parameter ${{\bf \alpha }} =$ (${{{{\bf \alpha }}}_1}, \ldots ,{{{{\bf \alpha }}}_{{\bf k}}})$, where ${{{{\bf \alpha }}}_{{\bf y}}}{\mathrm{\ }}( {1 \le {\mathrm{y}} \le {\mathrm{k}}} )$ was randomly selected from a binomial distribution ${{\bf B}}( {10y,\ 0.5} )$. For each clone of a proportion ${{\bf \rho }}$, somatic heterozygote mutations were generated at random genomic loci, with the total read-depth (${{{\boldsymbol{N}}}^{total}})$ randomly chosen from normal distribution $\mathcal{N}( \bf{125,8})$ and alternate allele count (${{{\boldsymbol{N}}}^{alt}})$ following binomial distribution ${{\bf B}}( {{{{\boldsymbol{N}}}^{total}} \times {{\bf \rho }}/2,0.5} )$, making sure that VAF follows distribution with mean VAF as ${{\bf \rho }}/2$. Then, false somatic variants were generated, the VAF of which were based on the reverse sigmoid function (eq. 8) to mimic the nature of real-world dataset.


(8) \begin{eqnarray*}\begin{array}{*{20}{c}} {P\left( {X = {{f}_{i.j}}{\mathrm{|}}{{v}_{i.j}} \in {\boldsymbol{c}}_{FV}^{\boldsymbol{A}}} \right) = \left( {1 - \frac{1}{{1 + \ {{e}^{\left( {100{{f}_{i.j}}\ - 5} \right)}}}}} \right) \times Constant} \end{array}\end{eqnarray*}

where ${\boldsymbol{c}}_{FV}^{\boldsymbol{A}}$ refers FV cluster of answer sets, and ${\boldsymbol{Constant}}$ is a constant to make the sum of probability density function 1. Mean and median VAF of the model were 0.029 and 0.027, respectively (see Supplementary Figure S3). Five different datasets, including a different number of false variants (0%, 2.5%, 5%, 7.5% or 10% of the total 500 variants), were evaluated accordingly.

Similarly, the SimData-lump dataset was generated by changing the shape parameter of the Dirichilet distribution ${{\bf \alpha }}{\mathrm{^{\prime}}} = ( {{{\bf \alpha }}_1^{\mathrm{^{\prime}}}, \ldots ,{{\bf \alpha }}_{{\bf k}}^{\mathrm{^{\prime}}}} )$, where ${{\bf \alpha }}_{{\bf y}}^{\mathrm{^{\prime}}}( {1 \le {\mathrm{y}} \le {\mathrm{k}}} )$ is randomly selected from a binomial distribution ${{\bf B}}( {10k,0.5} )$; this makes the clones more agglomerated. Likewise, five datasets with false variants added (0%, 2.5%, 5%, 7.5% or 10% of the total 500 variants) were prepared to evaluate the influence of false variants.

Here, $500 - {\mathrm{n}}( {{\boldsymbol{c}}_{FV}^{\boldsymbol{A}}} )$ somatic mutations were distributed to ${\mathrm{k}}$ clones, so the number of mutations per clone followed a multinomial distribution (${{\bf n}} = 500 - n( {{\boldsymbol{c}}_{FV}^{\boldsymbol{A}}} )$, ${{{{\bf p}}}_{\boldsymbol{i}}} = \frac{1}{k}$ for $i = ( {1,\ 2,\ \ldots ,k} )$). The VAF and number of mutations of each clone are depicted in Supplementary Figure S4. Base quality (BQ) of each variant was set to 20 (99% confidence).

Then, we expanded our benchmark by selecting the combination of total variants and mean read-depth among $Total\ variants = \{ {100,\ 500,\ 1000} \}$ and $Read\_depth = \{ {30,\ 125,\ 250} \}$, to verify the performance of CLEMENT according to the number of total variants and read-depths. In each dataset, we repeated the random sampling 30 times and evaluated the mean value.

#### Cell line mixture dataset (CellData)

CellData was constructed based on our previous study that provided 39 physical mixtures of six completely genotyped human cell lines (MRC5, RPE, CCD-18co, HBEC30-KT, THLE-2 and FHC) in various compositions (three or four cell lines out of the six) and cellular proportions (0.5–56%) (11). CellData consisted of fully diploid genomes by excluding sex chromosomes to ensure copy number neutrality.

In a mixture, cell line-specific variants form an individual clone. Also, variants that are shared between the cell lines form hypothetical ancestral clones. We downloaded the 1,100x whole-exome sequencing (WES) data of the 39 mixtures (M1-1 to M1-9, M2-1 to M2-12 and M3-1 to M3-18) from the Sequence Read Archive (SRA) repository database (SRP334852). Among the 39 mixtures, we used eight to construct the test sets. The exclusion criteria were as follows: (i) the presence of two uneven clones (clone size difference > 5 times) at inseparable frequencies (VAF difference $ \le 0.03$) (excluded M1-1,3,5,7 and M2-10,12), (ii) the presence of extremely small clones (clone size $ \le 30$ variants; mean clone size = 1629) (excluded M1-9, M2-1,3,5,7,9,11) and (iii) redundancy of the clone composition (all 18 M3 mixtures). As a result, eight test sets with 3–4 individual and 0–1 ancestral clone were prepared. A multi-sample dataset was generated by combining 2 or 3 samples, irrespective of mixture category (M1 or M2). Accordingly, 28 (=_8_C_2_) two-sample and 56 (=_8_C_3_) three-sample test sets were prepared. The characteristics of CellData, including clonal proportion, variants counts, and false variants compositions are listed in Supplementary Table S1.

For each WES dataset of the selected mixtures, somatic single nucleotide variants (SNVs) were selected using GATK MuTect2 (version 4.2.3.0) and filtered by FilterMutectCalls (ver. 4.2.3.0) with default parameters (22). SNVs that did not match any of the cell line genotypes were marked as false variants. Similar to SimData, we chose the total number of variants and the mean read-depth for the benchmark as 500 and 125, by downsampling the initially downloaded 1,100x datasets using picard (v2.26.4, http://broadinstitute.github.io/picard).

We extended our test datasets by applying 0 (0%), 13 (2.5%), 25 (5%), 38 (7.5%) and 50 (10%) false variants. Then, we generated the datasets without the ancestral clone and with one ancestral clone added. The number of mutations and mean read-depths for simulations were extended to a combination of $Total\ variants = \{ {100,\ 500,\ 1000} \}$ and $Read\_depth = \{ {30,\ 125,\ 250} \}$ to assess the performance of CLEMENT in various conditions. We conducted repetitive randomized trials (30 times) and comparisons for each dataset. In each trial, we selected variants through random sampling while maintaining the overall proportions of each clone. The test datasets for CellData are available on https://github.com/Yonsei-TGIL/CLEMENT.

#### Human normal tissue dataset (BioData)

BioData was prepared using a recent study that conducted whole-genome sequencing of 561 laser capture microdissected patches from 29 microscopic histological structures from three individuals (14). The number of clones from the 29 tissues was estimated by the genomic VAF and histological assessment in the original study and used in the test. We downloaded 524 732 somatic SNVs and VAFs from the 29 tissues (5 mono-clonal, 4 bi-clonal and 20 poly-clonal; according to the author's estimation) from the Supplementary Information of the study (14) and used them as inputs for testing. We discarded samples that did not pass the following conditions: (i) average read depth $ \ge 20$ and (ii) total number of mutations $ \ge 100$. Finally, we obtained 224 samples from 24 tissues to perform single-sample decomposition.

In the evaluation, mono- and bi-clonal samples from the original datasets were marked as $k$ (the number of clusters) $ = 1\ and\ 2$, respectively. For poly-clonal samples, a prediction of$\ k \ge 3$ was considered correct.

### Performance measurement

#### Scoring index

The test performance was measured in two terms, as follows: (i) the accuracy of the clone number estimation and (ii) the accuracy of variant membership. For (i), the deviation of the estimated clone number from the true number was scored in the root mean square error (RMSE) of all the trials (30 times). For (ii), the Adjusted Rand Index (ARI) (23) and membership score (${{{\boldsymbol{S}}}_{\boldsymbol{M}}}$) were used to performance measurement.

Let ${{{\boldsymbol{C}}}_{\boldsymbol{A}}} = \{ {{\boldsymbol{c}}_1^{\boldsymbol{A}},{\mathrm{\ }} \ldots ,{\boldsymbol{c}}_{{{\kappa }_A}}^{\boldsymbol{A}}} \}$ and ${{{\boldsymbol{C}}}_{\boldsymbol{P}}} = \{ {{\boldsymbol{c}}_1^{\boldsymbol{P}},{\mathrm{\ }} \ldots ,{\boldsymbol{c}}_{{{\kappa }_P}}^{\boldsymbol{P}}} \}$ be a set of clusters in answer and predicted output, and their indices as ${{{\boldsymbol{I}}}_{\boldsymbol{A}}} = {\mathrm{\ }}\{ {1,2, \ldots ,{{\kappa }_A}} \}$ and${\mathrm{\ }}{{{\boldsymbol{I}}}_{\boldsymbol{P}}} = \{ {1,2, \ldots ,{{\kappa }_P}} \}$. Then, we define ${{{\boldsymbol{u}}}_{i,j}}{\mathrm{\ }}( {1 \le i \le {{\kappa }_A},{\mathrm{\ }}1 \le j \le {{\kappa }_P}} )$ as the number of common variants in ${\boldsymbol{c}}_i^{\boldsymbol{A}} \in {{{\boldsymbol{C}}}_{\boldsymbol{A}}}{\mathrm{\ }}$and ${\boldsymbol{c}}_j^{\boldsymbol{P}} \in {\mathrm{\ }}{{{\boldsymbol{C}}}_{\boldsymbol{P}}}$. Also, let ${{{\boldsymbol{a}}}_i}$ and ${{{\boldsymbol{p}}}_j}$ be the number of variants in ${\boldsymbol{c}}_i^{\boldsymbol{A}}$ and ${\boldsymbol{c}}_j^{\boldsymbol{P}}$, respectively. ARI is defined as below:


(9) \begin{eqnarray*}\begin{array}{@{}*{1}{c}@{}} {ARI = \ \frac{{\mathop \sum \nolimits_{ij} \left( {\begin{array}{@{}*{1}{c}@{}} {{{{\boldsymbol{u}}}_{i,j}}}\\ 2 \end{array}} \right)\ - \ \left[ {\mathop \sum \nolimits_i \left( {\begin{array}{@{}*{1}{c}@{}} {{{{\boldsymbol{a}}}_i}}\\ 2 \end{array}} \right)\mathop \sum \nolimits_j \left( {\begin{array}{@{}*{1}{c}@{}} {{{{\boldsymbol{p}}}_j}}\\ 2 \end{array}} \right)} \right]/\left( {\begin{array}{@{}*{1}{c}@{}} n\\ 2 \end{array}} \right)\ }}{{\frac{1}{2}\left[ {\mathop \sum \nolimits_i \left( {\begin{array}{@{}*{1}{c}@{}} {{{{\boldsymbol{a}}}_i}}\\ 2 \end{array}} \right)\ + \ \mathop \sum \nolimits_j \left( {\begin{array}{@{}*{1}{c}@{}} {{{{\boldsymbol{p}}}_j}}\\ 2 \end{array}} \right)} \right]\ - \ \left[ {\mathop \sum \nolimits_i \left( {\begin{array}{@{}*{1}{c}@{}} {{{{\boldsymbol{a}}}_i}}\\ 2 \end{array}} \right)\mathop \sum \nolimits_j \left( {\begin{array}{@{}*{1}{c}@{}} {{{{\boldsymbol{p}}}_j}}\\ 2 \end{array}} \right)} \right]/\left( {\begin{array}{@{}*{1}{c}@{}} n\\ 2 \end{array}} \right)}}\ } \end{array}\end{eqnarray*}

where $n$ is the number of variants as forementioned.

However, for the test sets with false variants, ARI is not applicable due to its inability to discriminate the set of false variants from true clones. Symmetricity of ARI provides an advantage in not needing precise labeling of clusters, but there is also a limitation in its applicability when exact discrimination of FV is required. Therefore, we defined an additional score ${{{\boldsymbol{S}}}_{\boldsymbol{M}}}$ that measures the maximum matches of variant membership from all possible injection functions (between the answer and the predicted clusters) in answer set ${{{\boldsymbol{C}}}_{\boldsymbol{A}}}$ and the predicted set ${{{\boldsymbol{C}}}_{\boldsymbol{P}}}$.


(10) \begin{eqnarray*}\begin{array}{*{20}{c}} {{{{\boldsymbol{S}}}_{\boldsymbol{M}}} = \mathop {\max }\limits_{{\boldsymbol{R}} \in \mathcal{R}} \left\{ {\mathop \sum \limits_{\left( {i,j} \right) \in {\boldsymbol{R}}} {{{\boldsymbol{u}}}_{i,j}}} \right\} \times \frac{{100}}{n}} \end{array}\end{eqnarray*}

where ℛ is a set of relations ${\boldsymbol{R}} \subset {{{\boldsymbol{I}}}_{\boldsymbol{A}}} \times {{{\boldsymbol{I}}}_{\boldsymbol{P}}}$ satisfying that, if ${{\kappa }_A} \le {{\kappa }_P}$, then ${\boldsymbol{R}}$ is an injective function (${\boldsymbol{R}}\ :\ {{{\boldsymbol{I}}}_{\boldsymbol{A}}} \to {{{\boldsymbol{I}}}_{\boldsymbol{P}}}$ with $( {x,y} ) \in {\boldsymbol{R}}$ and $( {z,y} ) \in {\boldsymbol{R}}$ implies $x = z$), and if ${{\kappa }_P} \le {{\kappa }_A}$, then ${{{\boldsymbol{R}}}^{\boldsymbol{T}}}$ is an injective function, where ${{{\boldsymbol{R}}}^{\boldsymbol{T}}} = \{ {( {y,x} )\ :( {x,y} ) \in {\boldsymbol{R}}} \}$. To normalize it, we recalibrated by the number of variants, $n$. An example of the determination of ${{{\boldsymbol{S}}}_{\boldsymbol{M}}}$ is described in Supplementary Figure S5.

#### Testing of cancer decomposition tools

Three cancer decomposition tools were prepared for the test. PyClone-VI (version 0.1.1) was downloaded from the GitHub repository (https://github.com/Roth-Lab/pyclone-vi) and installed using Conda. SciClone (version 1.1.0) was downloaded and installed on R (version 4.2.0) following the installation instructions in (https://github.com/genome/sciclone). QuantumClone (version 1.0.0.9) was downloaded and installed on R (ver. 4.2.0) using CRAN (https://CRAN.R-project.org/package=QuantumClone). For SciClone and PyClone-VI, parameters for the copy number (‘major_cn’ and ‘minor_cn’) and tumor content (‘tumour_content’) were set to 1 for optimization. For QuantumClone, the ‘Genotype’ parameter was set to ‘AB’. Default values were used for all other parameters.

### Analysis in the real-world datasets

#### Mutational burden and signature analysis in bi- or poly-clonal samples

Out of a total 224 samples, CLEMENT identified 136 samples as monoclonal. For the remaining 88 bi- or poly-clonal samples, we measured the mutational burden in each clone. In cases where CLEMENT chose fuzzy clustering which does not provide binary membership, we assigned the membership of ${{v}_j}$ as $y$ where $\Theta ( {{{v}_j},{{c}_y}} )$ is maximized. We used standard deviation as a metric for dissimilarity of mutational burden. To determine the threshold where clones are not genetically identical, we employed a bootstrap approach with 100 times of iteration. The null hypothesis assumed that the mutational burden for each clone is the same. If the standard deviation of the proportions of each clone within the sample is beyond the 95% confidence interval (CI) of the distribution obtained through bootstrapping, we considered that sample to be highly dissimilar.

We analyzed the spectrum of mutational signatures in BioData following clonal decomposition using CLEMENT. For signature extraction and matrix formation, we utilized SigProfilerMatrixGenerator (https://github.com/AlexandrovLab/SigProfilerMatrixGenerator). Signature extraction, assignment to the COSMIC database, and visualization were performed using SigProfilerAssignment (version 0.0.29, https://github.com/AlexandrovLab/SigProfilerAssignment) (24). We specifically focused on SBS1, 2, 4, 5/40, 7a, 7b, 13, 16, 18, 32, 35, 88 and 91, as outlined in Moore *et al.*’s paper (14), to maintain the pattern of signatures from the original paper. Notably, we combined SBS5 and SBS40 into SBS5/40, following the previous work. We explored the percentage of SBS1 and SBS5/40 in each clone, as discrepancies among tissues were noted in the previous study.

#### Clonality analysis in adrenal glands

We obtained three layered tissues (Zona Glomerulosa (ZG), Zona Fasciculata (ZF) and Zona Reticularis (ZR)) from one donor (PD28690) at five different regions (L1–L5), resulting in a total of 15 samples. One-sample and two-sample clonal decompositions were performed using CLEMENT, PyClone-VI, SciClone and QuantumClone.

We performed unsupervised hierarchical clustering and visualization between the samples using scipy (ver. 1.10. https://docs.scipy.org/doc/scipy/reference/generated/scipy.cluster.hierarchy.dendrogram.html) based on Jaccard similarity. By comparing the mutation sets and decomposed clones using CLEMENT, we analyzed the clonality in two adjacent tissues (ZG – ZF). We used BioRender (http://app.biorender.com/) to create the illustration.

## Results

### Clonal decomposition in simulated data

**Figure 2.**
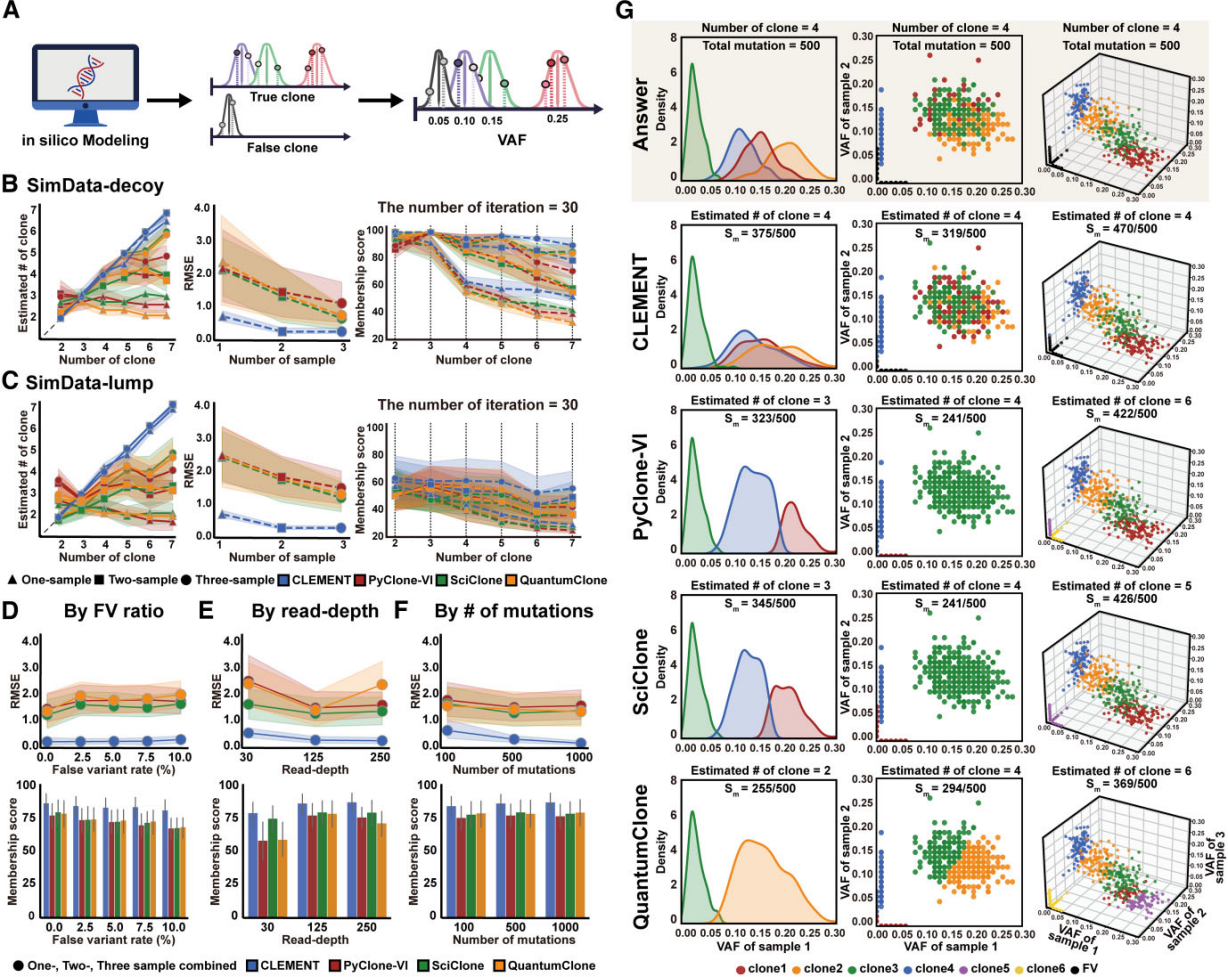
Test on simulated data. (**A**) Establishment of an *in silico* mixed non-tumor diploid model using Dirichlet's random sampling. (B, C) Performance comparison for estimated number of clones (left), RMSE (middle) for number of clones, and membership score (${{{\boldsymbol{S}}}_{\boldsymbol{M}}}$, right) with 500 mutations and mean depth 125 in (**B**) SimData-decoy without false variants, (**C**) SimData-lump without false variants. Extended benchmark of SimData-decoy without false variants by (**D**) false variant ratio, (**E**) read-depth and (**F**) the number of mutations. Each point represents the mean value of 30 times of iteration, and 95% confidence interval (CI) is depicted as a shadow. (**G**) Examples of one-sample (left), two-sample (middle), and three-sample (right) decomposition that show the superiority of CLEMENT. False variants are depicted as black dots. FV: false variants, VAF: variant allele frequency, RMSE: root mean square error.

We tested CLEMENT on simulated data sets (SimData-decoy and SimData-lump) over various conditions (Figure 2a). The primary SimData is a collection of 18 simulated samples consisting six different numbers of clones ($k$ = 2, 3, 4, 5, 6, and 7), and three different numbers of samples ($m$ = 1, 2, and 3) containing 500 variants ($n$ = 500) whose mean read-depths are 125, without the embedment of false mutations (see Materials and metthods). The mean VAFs of each cluster exhibited divergence in SimData-decoy and agglomerated in SimData-lump, although the difference was narrowed as the number of clones increased (Supplementary Figure S4). Three cancer decomposition tools (PyClone-VI, SciClone and QuantumClone) were also tested on the datasets and compared with CLEMENT.

Firstly, in the SimData-decoy dataset, we observed a nearly perfect correlation between the estimated number of clones and answers in CLEMENT (Figure 2b, left). Specifically, we observed a remarkable improvement in terms of RMSE (0.06–0.55 versus 0.96–2.09, 0.58–2.27 and 0.48–2.03 in CLEMENT versus PyClone-VI, SciClone, and QuantumClone, respectively; ranges within one-, two-, and three-sample datasets, Figure 2b, middle) and the membership agreement metrics (27.0–58.3% and 28.8–40.0% increase in membership score ${{{\boldsymbol{S}}}_{\boldsymbol{M}}}$ and ARI, respectively; ranges within one, two, and three-sample datasets, Figure 2b, right). The improved accuracy was intensified with the increase in sample and clone numbers. In particular, CLEMENT maintained its performance, while all the cancer decomposition tools showed severe underestimation of clone numbers in $k \ge 4$ (Figure 2B, right).

We also observed the superiority of CLEMENT compared to the other tools in the SimData-lump dataset, in which the VAF values of mutations were more condensed, making clustering more challenging. CLEMENT demonstrated superior performance in estimating the number of clones, achieving nearly perfect matches for each clone (Figure 2C, left). In terms of RMSE, CLEMENT exhibited better results ranging 0.05–0.49, compared to 1.33–2.37, 1.12–2.36 and 1.01–2.31 in PyClone-VI, SciClone and QuantumClone, respectively (Figure 2C, middle). Additionally, membership agreement to clones was higher in SimData-lump (40.2–60.2% higher in ${{{\boldsymbol{S}}}_{\boldsymbol{M}}}$ and 43.1–53.3% higher in ARI, Figure 2C, right, Supplementary Table S2). Similar to that in SimData-decoy, the superiority of CLEMENT over other tools was remarkable as the number of clones increased (11.3–33.9% higher in $k = 2$ and 38.5–60.2% higher in $k = 7$ regarding ${{{\boldsymbol{S}}}_{\boldsymbol{M}}}$). Additionally, the accuracy of CLEMENT was even more significant as more samples were included. Especially in $m = 3$, the performances were sustained even when more clones were introduced where accurate decomposition was harsher.

We extended our evaluations of the SimData-decoy and SimData-lump datasets under various conditions, including changing the ratio of inserted false variants, read-depths, and the number of mutations (Figure 2D–F, Supplementary Figure S6a–c). CLEMENT consistently outperformed the other tools across all conditions, showing particularly reliable outputs in estimating the number of clones. The superiority of CLEMENT became more pronounced with an increase in the higher read-depths (11.8–35.4% higher in ${{{\boldsymbol{S}}}_{\boldsymbol{M}}}$ and 1.10–2.14 in $\Delta$RMSE), greater number of mutations (11.6–13.6% higher in ${{{\boldsymbol{S}}}_{\boldsymbol{M}}}$ and 1.17–1.44 in $\Delta$RMSE), and more embedded false variants (11.8–12.0% higher in ${{{\boldsymbol{S}}}_{\boldsymbol{M}}}$ and 1.24–1.70 in $\Delta$RMSE). CLEMENT successfully isolated false variant cluster in 65.2% of whole simulations, whereas the other tools erroneously allocated the false variants into clusters or considered them as true biologic clone. Identification of false variant clusters became more feasible when more samples were provided (see Supplementary Table S3).

A more detailed inspection of the predicted cluster compositions provides a better understanding of the underlying characteristics of CLEMENT and cancer decomposition algorithms (Figure 2G). In the presence of multiple clones with overlapping VAF ranges, cancer decomposition tools are more inclined to predict a larger, merged cluster instead of smaller individual clones, resulting in an underestimation of clone numbers. The strong feature of the CLEMENT is its superior performance in more agglomerated datasets that reflect real-world biology, via fuzzy clustering.

### Test on *in vitro* cell line data

**Figure 3.**
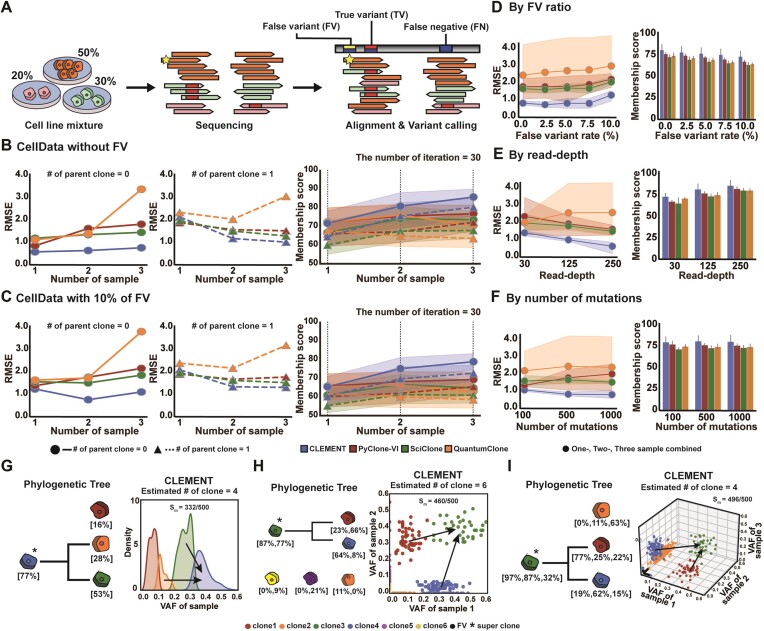
Test on pre-genotyped mixed cell line data. (**A**) Illustrative diagram of analysis utilizing mixed cell line data. (**B**, **C**) Comparison of RMSE (left and middle) for the number of clones and mean membership score (${{{\boldsymbol{S}}}_{\boldsymbol{M}}}$, right) with 95% confidence interval (CI, shadow) in one-sample (*n*= 8), two-sample (*n*= 28), and three-sample (*n*= 56) cell line datasets with (B) no false variant or (C) 10% false variants. Extended benchmark by (**D**) FV ratio, (**E**) read-depth and (**F**) the number of mutations are also described. (**G–I**) Phylogeny reconstruction of superclone-added cell line data in one-sample input (G, M1-6), two-sample input (H, M1-8 + M2-4) and three-sample input (I, M1-4 + M1-6 + M1-8). False variants are depicted as black dots. FV: false variants, VAF: variant allele frequency, RMSE: root mean square error.

We tested CLEMENT on another test set (CellData) constructed from mixtures of pre-genotyped cell lines in various proportions (Figure 3A, see Materials and methods for details). Unlike the simulation datasets, CellData contains a series of false negatives when performing two-sample or more decomposition because low VAF mutations are easily missed in single-sample calling (Supplementary Figure S7). Additionally, several samples in CellData consists of different type of mixtures (e.g. M1-2 of M1 and M2-4 of M2, see Supplementary Table S1), resulting in a series of sample-restricted clones that reflect real-world biology. Therefore, direct sequencing of physical clones and conventional variant calling offers a most realistic scenario for non-tumor decomposition. Like the SimData dataset test, CLEMENT showed superior performance.

In the absence of false variant and ancestral clones, CLEMENT exhibited better performance in both RMSE and ${{{\boldsymbol{S}}}_{\boldsymbol{M}}}$. CLEMENT demonstrated the highest accuracy in estimating clone numbers, particularly in three-sample decomposition (RMSE = 0.58–0.78) (Figure 3b, left). Conversely, cancer decomposition tools either under- or over-estimated clone numbers, resulting in significantly higher RMSE values (0.85–1.79, 1.18–1.43 and 1.11–3.34 in PyClone-VI, SciClone and QuantumClone, respectively; ranges within one-, two- and three-sample datasets). Additionally, when one superclone was added, CLEMENT demonstrated the best performance, except for one-sample decomposition (Figure 3B, middle). We speculate that this is because most of the clones in CellData have extremely low prevalence, making their superclone challenging to discern in one-sample decomposition. Generally, the accuracy of CLEMENT improved as more samples were provided, whereas the other tools exhibited inconsistency. CLEMENT also showed the superiority in terms of ${{{\boldsymbol{S}}}_{\boldsymbol{M}}}$ regardless of the presence of a superclone, although the differences among the tools were not as significantly pronounced as those in SimData (∼7.4%, ∼9.1% and ∼21.2% higher for one-, two- and three-sample decomposition, Figure 3b, right). The same phenomenon was observed in terms of ARI (Supplementary Table S4)

In the presence of false variant data, ranging from 2.5% to 10% of the entire datasets, CLEMENT outperformed other cancer decomposition tools significantly. When 10% false variants were added, CLEMENT showed the best RMSE and ${{{\boldsymbol{S}}}_{\boldsymbol{M}}}$ for all sample numbers (Figure 3C). Its superiority over the other cancer decomposition tools was more pronounced than that in the absence of false variants (∼7.9%, ∼14.2% and ∼26.7% higher for one-, two- and three-sample decomposition). All the tools tended to be less accurate when more false variants were included, whereas CLEMENT maintained relative overall performance (Figure 3D). CLEMENT successfully identified false variant data in two- and three- sample test sets (∼29.5 and ∼49.1% detection rate, respectively, Supplementary Table S5). In one-sample data, discrimination of false variants was challenging because the mean VAF of false variants (0.029, see Supplementary Figure S3) made identification alongside other low VAF clones (0.01–0.04) extremely unrealistic.

In the extended test sets that include various combinations of read-depths and the number of mutations, CLEMENT generally outperformed the other tools (Figure 3E, F, Supplementary Figure S8a, b). In 30× downsample and 100 mutations datasets, the superiority of CLEMENT was not as remarkable as that in other conditions, because the densely populated clones in low VAF of CellData are easily influenced by the harsh condition, making decomposition much more challenging. Especially, among the 30× data, variants of low VAF were filtered in the variant calling step, distorting the input information and resulting in right-shifting of the clusters. However, CLEMENT still maintained competitiveness compared to the others and outperformed them in all the other tests.

When a superclone was added to CellData, CLEMENT appropriately reconstructed superclone-subclone structures. The examples of reconstructed clone structures confirmed the accurate discrimination of ancestral clones from individual ones in various conditions, which was consistent with the answer datasets (Figure 3G: one-, 3H: two-, 3I: three-samples).

### Test on human multi-clonal normal tissues

**Figure 4.**
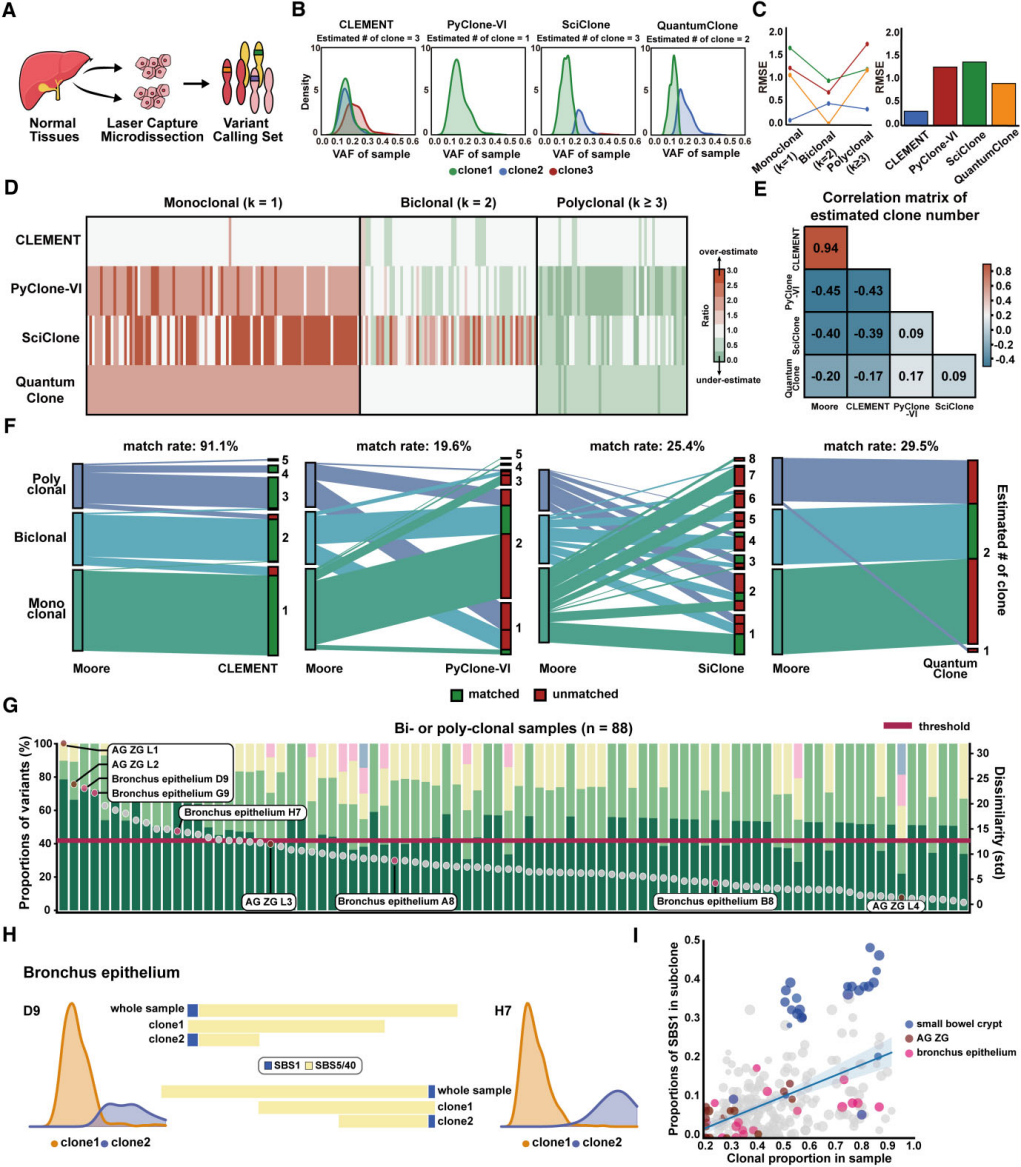
Test on human microdissected tissue data. (**A**) Schematic figure for obtaining clonal data in normal tissue, which shows high genomic similarity. (**B**) Confirmation of successful separation of CLEMENT through one-sample decomposition example (PD43850-pancreas duct-D7). (**C**) RMSE in each clonality (mono-clonal, bi-clonal, and poly-clonal) for four decomposition methods (left). Barplot (right) describes the RMSE for all samples. (**D**) Heatmap depicting the relationship between Moore *et al.*’s conjecture and other tools. Red indicates an overestimation of the number of clones compared with Moore's, and green refers to underestimation. (**E**) Correlation matrix and coefficients of estimated number of clones between the tools, including Moore *et al.*’s conjecture. (**F**) Alluvial plots for the number of estimated clones between Moore's conjecture and decomposition tools. (**G**) Stacked bar plot depicting the proportions of subclones for 88 bi- or poly-clonal samples. Circle indicates the dissimilarity of number of mutations calculated by standard deviation. (**H**) Composition of mutational signatures in two bronchus epithelium tissues. (**I**) Correlation was noted between clonal proportion in sample and proportions of SBS1 in each subclone. Linear regression was plotted as blue line (*r* = 0.49). VAF: variant allele frequency.

Finally, we applied CLEMENT to the sequencing of 24 microdissected human normal tissues that showed mono- to poly-clonal microstructures in Moore *et al.* (12) (BioData) (Figure 4A). The number of clones identified in the original study based on genomic profiling and histological assessment served as the gold standard. Clonality analysis was performed based on a sample level that evaluated the predicted number of clones. Only CLEMENT provided the fuzzy clustering that reflected the agglomerated nature of human data (Figure 4B). In total, CLEMENT estimated the exact clone numbers in 204 samples (91.1%) out of 224, with an RMSE of 0.30, outperforming the other tools (# exact match = 44 (19.6%), 57 (25.4%), and 66 (29.4%); RMSE = 1.12 (0.84–1.51), 1.45 (0.91–1.86) and 0.85 (0.0–1.03) in PyClone-VI, SciClone, and QuantumClone, respectively) (Figure 4C, D). We found a high correlation (Pearson's *r* = 0.94) between the predicted numbers and the gold standard in CLEMENT, whereas almost no correlation (r = -0.45–-0.20) was observed in the three cancer decomposition tools (Figure 4E). Notably, QuantumClone converged to bi-clonality in nearly all samples, which clearly demonstrated the weakness of the Bayesian Information Criterion (BIC) method when determining the number of clones (Figure 4F). Conversely, SciClone tended to produce a large number of clones in nearly all cases, indicating that the RMSE of SciClone in poly-clonal ($k \ge 3)$ tissues were erroneously overestimated.

For bi- or poly-clonal samples (*n* = 88, Supplementary Figure S9), we investigated the genomic profile for each clone and found dissimilar features within the clones. First, we examined the evenness of the number of mutations among the clones by calculating the standard deviation. We observed discrepancies in clonal mutational burden distribution among the samples (Figure 4G). The dissimilarity was well observed in samples with multiple peaks. For example, in adrenal gland zona glomerulosa (ZG) L1, the green clone included most of the mutations, whereas two condensed clones (light green and beige) had fewer mutations. Clones in normal samples have long been thought to be homogenous, but we witnessed that some populations (18%) exhibited distinguished discrepancies beyond the threshold, necessitating precise clonal decomposition. Additionally, we found that the dissimilarity varied even within the same tissue, such as ZG and bronchus epithelium. Clonal inference (proportions and number of clones) in normal tissue bulk datasets has been made based on the assumption that all clones are homogeneous, but we suggest clonal inference after exact clonal decomposition.

Next, we investigated the pattern of mutational signatures at the clone-level. The average number of mutations per clone was 415, which is sufficient to decompose the mutational signatures according to the genomic context. SBS5/40 was the dominant mutational pattern in most tissues, except for the small bowel crypt where SBS1 was the major mutational pattern. The discrepancy among the tissues was mentioned in a previous publication (14), and we reaffirmed the same phenomenon at the clone-level. Interestingly, we found that bronchus epithelium showed a different pattern of mutational signatures between the clones. For example, bronchus epithelium H7 and D9, which showed a clear bimodal peak and were decomposed by CLEMENT as bi-clonal, were divided as a major clone with SBS1 and a minor clone consisting only SBS5/40 (Figure 4h). We expanded our inspection for all clones of all samples. We noted the positive relationship between the clonal prevalence and the proportion of SBS1 (correlation coefficient *r* = 0.49, Figure 4I). As clones with higher VAFs indicate mutations occurred earlier (25), we hypothesized that the pattern of mutational signatures differs by the timing of mutations acquisition. A single-cell genome sequencing of the forebrain revealed the C > T mutations are enriched in early mutations (26), supporting our finding in that SBS1 signature mostly represents C > T mutations. Although direct evidence of high allelic fraction clones possessing high stemness is limited, we offer a glimpse of the relationship between mutational contexts and developmental dynamics at the clone level.

### Analysis on microscopic tissue of adrenal gland cortex

**Figure 5.**
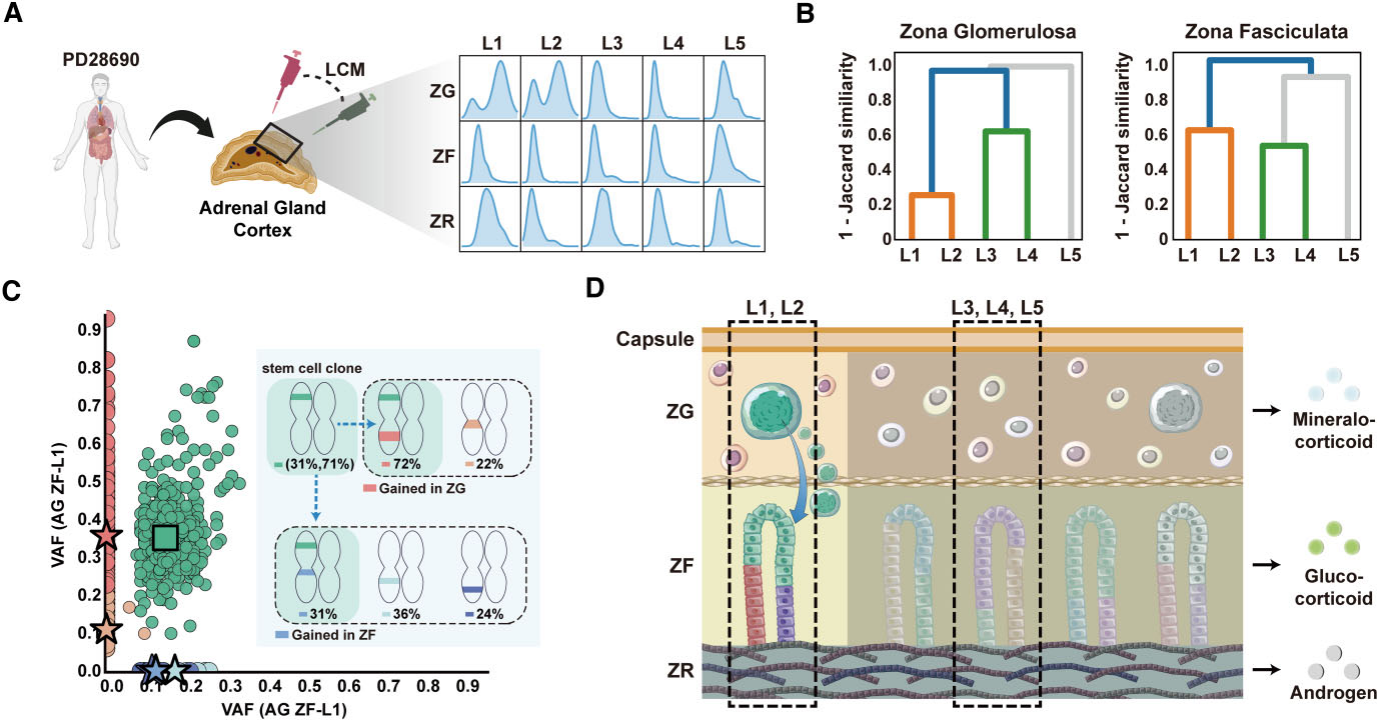
Clonality analysis of adrenal gland cortex. (**A**) Further adrenal gland analysis in 15 tissues. The blue figures show the distribution of variant allele frequency (VAF). (**B**) Unsupervised hierarchical clustering based on Jaccard similarity in ZG and ZF. (**C**) Two-sample decomposition revealing the presence of superclone, indicating the adult stem cell in adrenal gland cortex. (**D**) Proposed concepts of clonal migration in the adrenal gland. Large blue cell implies an adult stem cell in ZG. ZG: Zona Glomerulosa, ZF: Zona Fasciculata, ZR: Zona Reticularis Adapted from ‘Organs, multiple systems’, ‘Pipette (symbol)’ and ‘Adrenal Gland Structure and Hormones Production’, by BioRender.com (2024). Retrieved from https://app.biorender.com/biorender-templates.

Further analysis of 15 adrenal gland tissues revealed the heterogeneous nature of clonal compositions (Figure 5a). Cortex of adrenal glands consists of three layers—Zona Glomerulosa (ZG), Zona Fasciculata (ZF) and Zona Reticularis (ZR)—from the outer to the inner layer, each producing different steroid hormones. Since 1883, the presence of adult stem cells in the periphery of the adrenal gland cortex and formation of the ZG–ZF axis have been hypothesized (‘centripetal migration model’) (27), which was validated through BrdU staining (28).

In BioData, we observed that the outermost layer, ZG of L1 and L2 had definite dual peaks, implying more than bi-clonality. Conversely, ZF of L1 and L2 had a single peak, suggesting highly agglomerated poly-clonal tissues. Jaccard similarity within ZG and ZF revealed that L1 and L2 were genetically equivalent, as well as L3 and L4 (Figure 5B, Supplementary Figure S10). We noted substantial shared mutations between ZG-L1 and ZF-L1 (Jaccard similarity = 0.38), whereas there was no shared mutation between ZG-L3 and ZF-L3 (Jaccard similarity = 0.0, Supplementary Figure S11). Interestingly, VAFs of shared mutations were similar to the dominant peak in ZG (0.38) and one of the homogenous clones in ZF (0.17), indicating the clonality between the ZG-L1 and ZF-L1. In a two-sample decomposition (Figure 5c), CLEMENT revealed the presence of a superclone that has the major clone of ZG-L1 and one of the homogenous poly-clonal backgrounds in ZF-L1 as its subclones. From these observations, we confirmed the presence of clonality in ZG-L1 and ZF-L1, indicating superclone-subclone structures. In normal tissue, a superclone in localized tissue is equivalent to the adult stem cell. Therefore, we concluded that the adult stem cell clone resides in the adrenal gland, migrates to ZG and ZF and proliferates. This clone is clearly distinguished from the poly-clonal background in ZF-L1, which seems to be multifurcated from the developmental period. Conversely, in L3, there was no clonality between ZG and ZF, indicating the absence of an adult stem cell clone. The absence of clonality in ZG-L3 and ZF-L3 supports independent clear zonation in the developmental stage (27). These findings are in accordance with previous findings that postmeiotic stem cells are found in *localized* areas of ZG (29) (Figure 5D). Throughout the entire process, we took advantage of CLEMENT, which provided (i) clonal reconstruction and (ii) homogenous poly-clonality in most samples, unlike the other tools. Clonality analysis in ZG-L5 or ZR were unavailable due to the limited number of mutations and extremely low cellular fractions (Supplementary Figure S12).

## Discussion

In this study, we pioneered a novel method of genomic decomposition in non-tumor samples. The EM-based algorithm with additional strategies for the proper handling of non-tumor sequencing data led to a substantial improvement in estimating the clone composition and structures and was validated in three independent test sets. In-depth analysis under various conditions, including the presence of false variants and different inter-clone similarities confirmed the effectiveness of the strategies, as well as the limitation of current cancer decomposition tools in normal clone analysis.

Recent efforts in identifying clonal heterogeneity (9,30,31) and developmental lineage (6,32) in normal tissues identified essential characteristics of non-tumor subclones in multiple aspects, especially against traditional cancer-derived samples. While the post-embryonic somatic and mosaic mutations are the major sources for both subclones, the differences in mutation rates (1–100 per Mb in cancer versus <1 per MB in normal tissues), mutation types (frequent CNAs and chromosomal instability in cancer), VAFs (very low in normal tissues), and the colonization path (fast clonal expansion in cancer versus slow to no clonal expansion in normal tissues) confer the intrinsic differences between their subclones, which set the basis for our study. In addition to the genomic properties that are already formulated in CLEMENT (false variants, clone similarity, and absence of CNAs), more sophisticated features can also be employed for further improvement, such as the distribution of VAFs within a clone and the mutation signatures.

As in the tumor decomposition, there is a growing interest in the use of non-tumor clonal analysis to understand disease pathogenesis. For example, the existence of the stem cell niche and the regeneration of the cells *in situ* has been widely studied to assume pathogenic clones in normal tissues, as shown in the colonic crypt, esophagus epithelium, and the subventricular and subgranular zone of the brain (33,34). In addition, recent studies adopt a new perspective on genomic regeneration and clonal evolution in investigating neurodegenerative diseases, including Alzheimer's disease (35), hippocampal sclerosis (36), and schizophrenia (37). As we discovered the localized stem cell in the adrenal gland cortex, understanding the clonal structure using appropriate clonal decomposition may be greatly helpful in expanding the knowledge of normal or non-tumor tissue. We expect that CLEMENT will provide a better assessment of the compositions and microscopic structures, as well as the number, dispersion, and phylogeny of these clones.

Despite the substantial achievements of CLEMENT, there are a few remaining technical issues to be resolved. First, the robust mathematical background for the modified E-M algorithm applied in CLEMENT has not been well discussed. Unlike the orthogonal E-M algorithm, CLEMENT interrupts the iteration if the *subclone rule* or *sum rule* is unsatisfied. Additionally, CLEMENT employs multiple probabilistic models in the E step considering FP and FN, to reflect real-world biology. However, we observed the convergence of E–M iteration and the achievement of optimal results by simulating more than thousands of datasets (Supplementary Figure S13). Second, proper discrimination of false variants from true low-allele frequency mutation is still a challenging problem. We noted that significant portions of false variants were not properly identified, especially in a single-sample case. We expect that the incorporation of recent technical advances, such as duplex sequencing (38) or a bioinformatics approach (39), could address the problem. Third, performance is limited when low read-depth is provided because of a lack of read information to be classified as multiple clones, and uncalled or filtered low VAF mutations distorting the clonal structure, resulting in the right-shifting of low prevalent clones. We hope that further advance in biotechnology and computational algorithms will improve the forementioned problems.

## Supplementary Material

gkae527_Supplemental_File

## Data Availability

CLEMENT with all the code and data used in this manuscript is available at the FigShare repository (https://figshare.com/s/12f35ff0785ad5c27921) and GitHub repository (https://github.com/Yonsei-TGIL/CLEMENT).
