# Supplementary material for: CLEMENT: genomic decomposition and reconstruction of non-tumor subclones
Source: Nucleic Acids Res. 2024 Jun 26;52(14):e62. doi: 10.1093/nar/gkae527 (PMC11317173; doi:10.1093/nar/gkae527)
Supplement: gkae527_Supplemental_File [file gkae527_supplemental_file.pdf]

Supplementary Data

CLEMENT: GENOMIC DECOMPOSITION AND RECONSTRUCTION OF NON-TUMOR SUBCLONES

Young-soo Chung[1, †], Seungseok Kang[1, †], Jisu Kim[2, 3], and Sangwoo Kim[1, *]

[1]Department of Biomedical Systems Informatics, Brain Korea 21 PLUS Project for Medical Science, Yonsei University College of Medicine, Seoul, 03722, Republic of Korea
[2]DataShape team, Inria Saclay Île-De-France, Palaiseau, 91120, France
[3]Department of Statistics, Seoul National University, Seoul, 08826, Republic of Korea

† These authors contributed equally to this work.
* To whom correspondence should be addressed.
Tel: (82)-2228-2589
Fax: (82)-2227-8308
Email: swkim@yuhs.ac

Contents

Supplementary Figures 3
Figure S1. (Method) Definitions of subclone and superclone 3
Figure S2. (Method) Distinguishing the individual clone and ancestral clone 4
Figure S3. (Method) Distribution of false variants. 5
Figure S4. (Method) Clonal proportions in SimData. 6
Figure S5. (Method) A descriptive example of the measuring of the membership score ($\boldsymbol{SM}$). 7
Figure S6. (SimData) Benchmark of each decomposition tool in various conditions. 8
Figure S7. (CellData) Illustration of false positives and false negatives. 9
Figure S8. (CellData) Benchmark of each decomposition tool in various conditions. 10
Figure S9. (BioData) Clonal decomposition for bi- or poly-clonal samples of human microdissected normal tissues. 11
Figure S10. (BioData) Jaccard similarity between L1–L5 locations within each zone. 12
Figure S11. (BioData) One-sample decomposition by CLEMENT in adrenal gland and shared mutations between ZG and ZF in five locations (L1–L5). 13
Figure S12. (BioData) Basic statistics for 15 adrenal gland tissues. 14
Figure S13. (Discussion) Convergence of CLEMENT by iteration. 15
Supplementary Tables 16
Table S1. (Method) Characteristics of CellData (clonal proportions and number of total variants). 16
Table S2. (SimData) Demonstration of the superiority of CLEMENT over other cancer decomposition tools (PyClone-VI, SciClone, and QuantumClone) in terms of the Adjusted Rand Index (ARI) in various situations. 17
Table S3. (SimData) Detection rate of false variants in various situations. 18
Table S4. (CellData) Demonstration of the superiority of CLEMENT over other cancer decomposition tools (PyClone-VI, SciClone, and QuantumClone) in terms of the Adjusted Rand Index (ARI) in various situations. 19
Table S5. (CellData) Detection rate of false variants in CellData. 20

Supplementary Figures

Figure S1. (Method) Definitions of subclone and superclone

(a) Subclone refers to a clone that acquires subclonal mutations from the common progenitor clone, superclone. The sum of proportions of subclones should be 1. (b) In a complex phylogenetic tree of clones, subclones are defined at several levels. All the sets in the diagram can be a set of subclones.

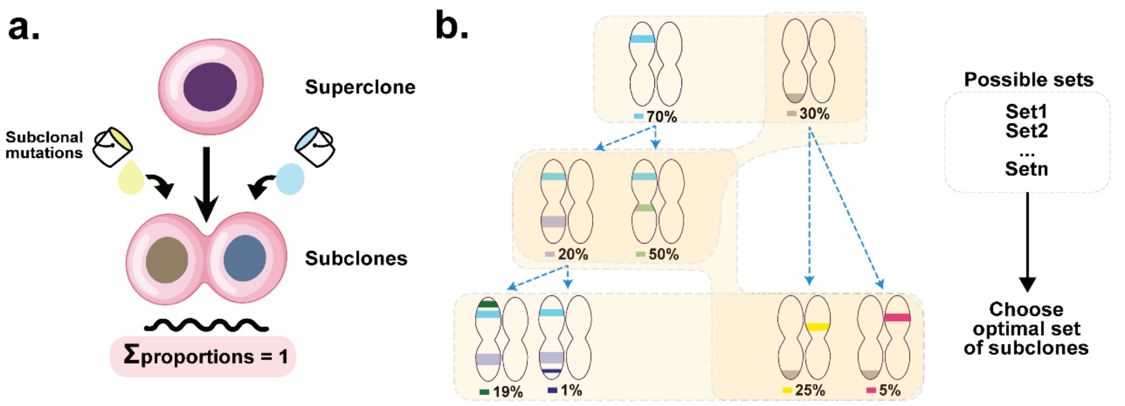

Figure S2. (Method) Distinguishing the individual clone and ancestral clone.

(a) Illustration of successful clustering and reconstruction of superclone-clone relationship. c_1, c_2, and c_3 comprises C^{ind}, and c_4 is an ancestral clone of c_1 and c_2. (b) Example of unacceptable clustering result because c_5 is neither element of C^{ind} nor C^{anc}.

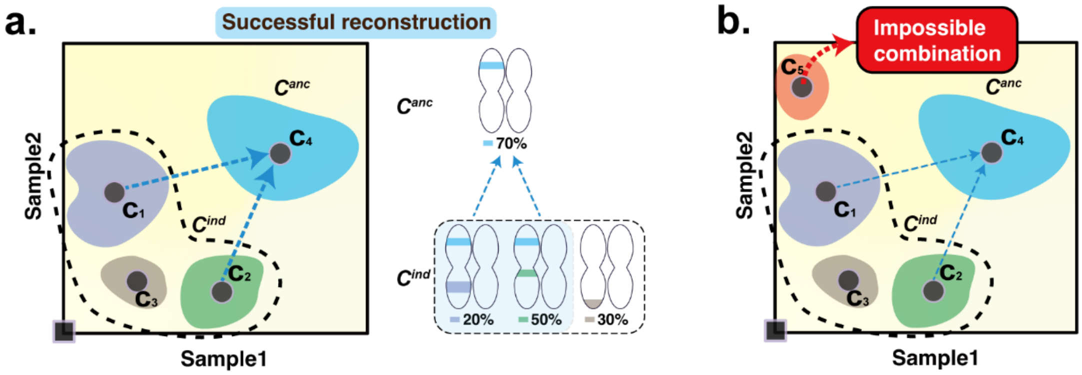

Figure S3. (Method) Distribution of false variants.

VAF distribution of false variants in (a) SimData and (b) CellData.

VAF: Variant Allele Frequency

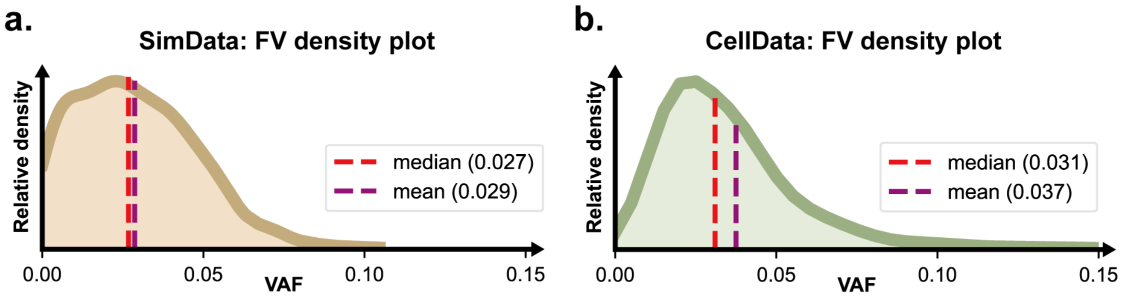

Figure S4. (Method) Clonal proportions in SimData.

(a) Mean VAF of each clone and (b) the number of mutations of each clone according to number of clones in one-sample SimData. 30 random datasets per each condition were generated. Gray square refers false variant cluster.

VAF: Variant Allele Frequency

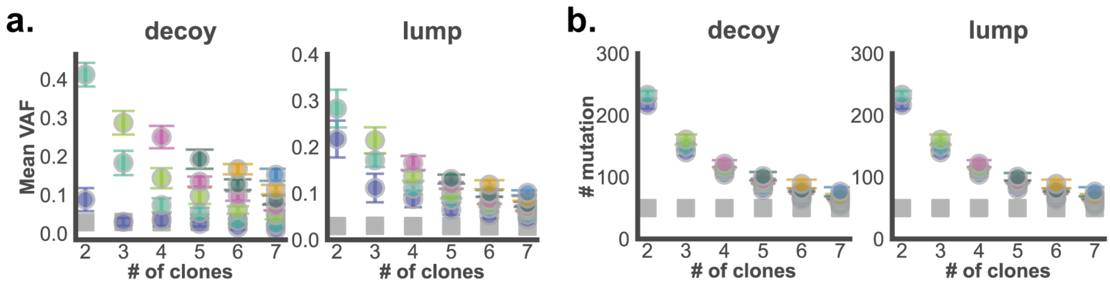

Figure S5. (Method) A descriptive example of the measuring of the membership score (S_M).

(a) An example of the answer set (left) and predicted set by any decomposition algorithm (right) given a two-sample dataset. The membership score (S_M) would be 11 if matched perfectly. (b) $u_{i,j}$, the number of common variants between two clusters, is described (left), and the calculation process is shown (right).

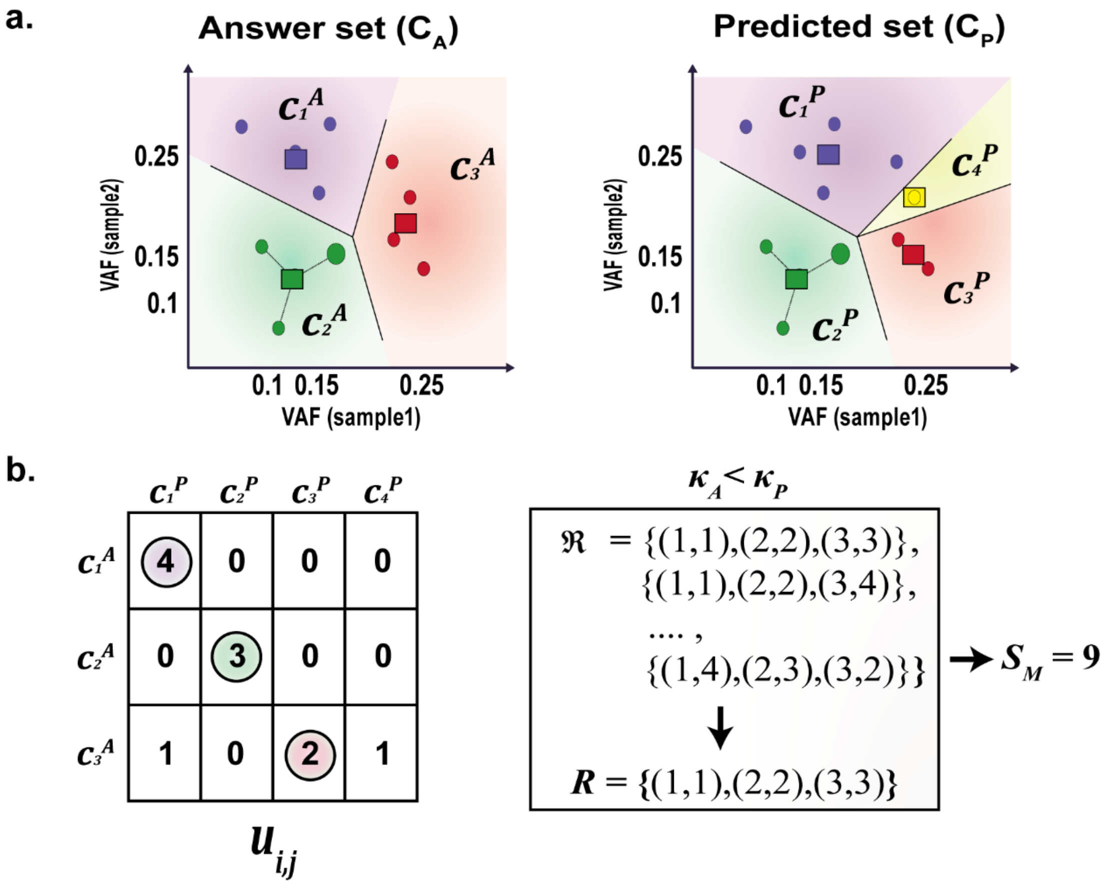

Figure S6. (SimData) Benchmark of each decomposition tool in various conditions.

RMSE for clone number estimations (left) and mean membership score (S_M, right) by (a) the read-depth, (b) number of mutations, and (c) false variant (FV) ratio.

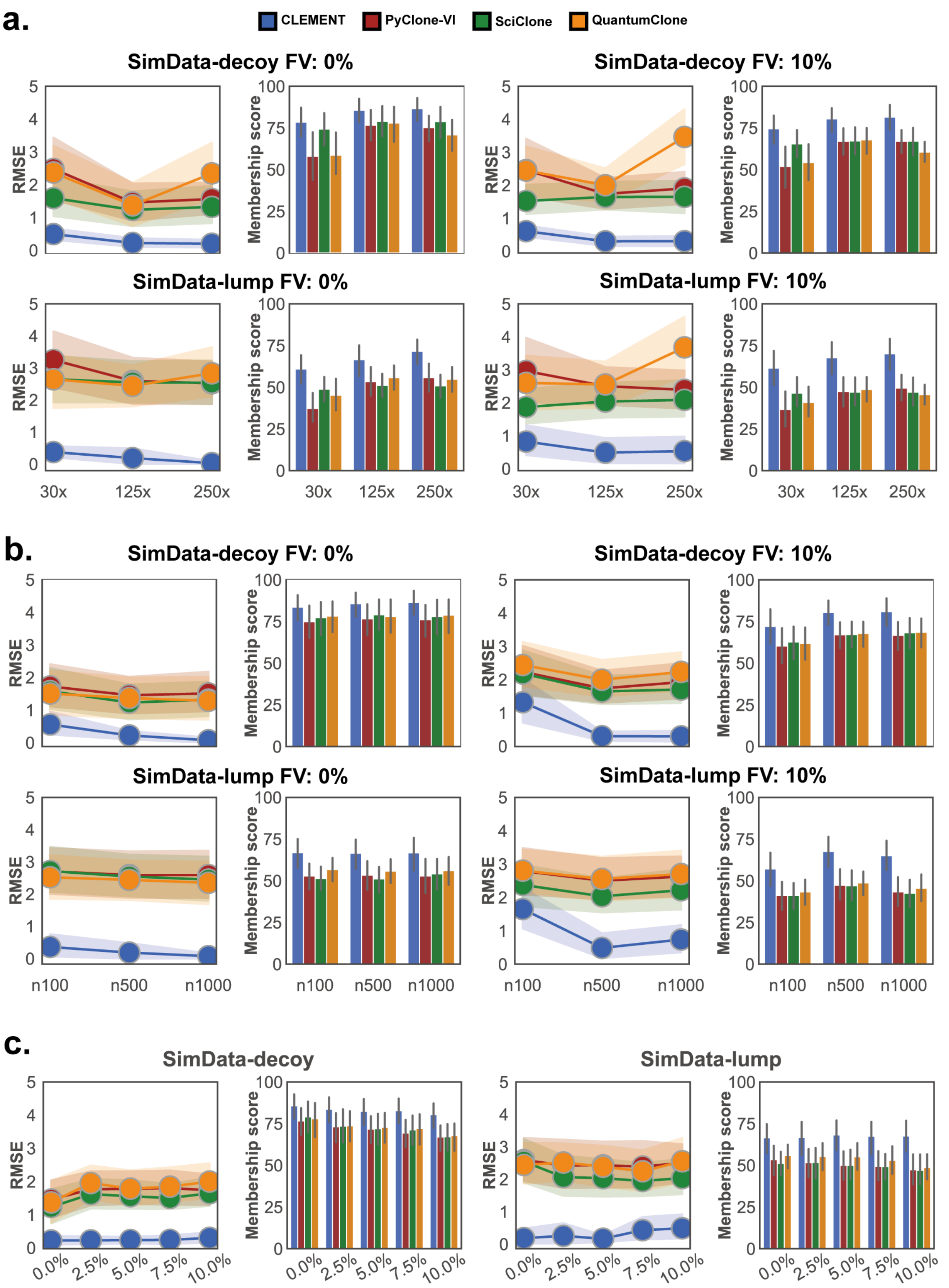

Figure S7. (CellData) Illustration of false positives and false negatives.

VAF: Variant Allele Frequency

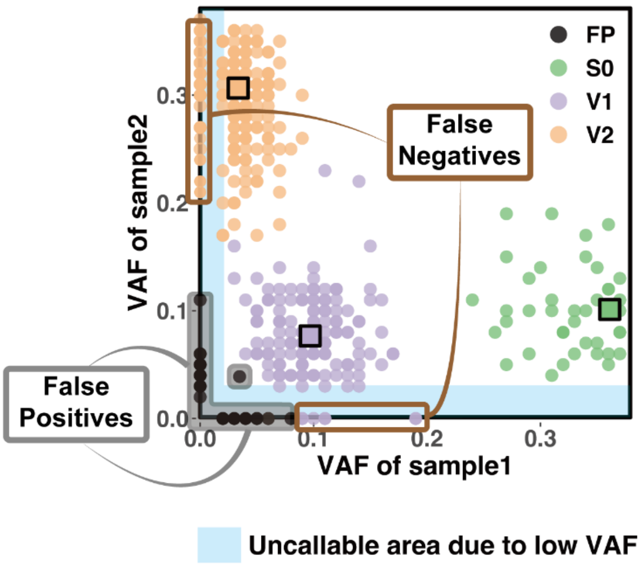

Figure S8. (CellData) Benchmark of each decomposition tool in various conditions.

RMSE for clone number estimations (left) and mean membership score (S_M, right) by (a) the read-depth, (b) number of mutations, and (c) false variant (FV) ratio.

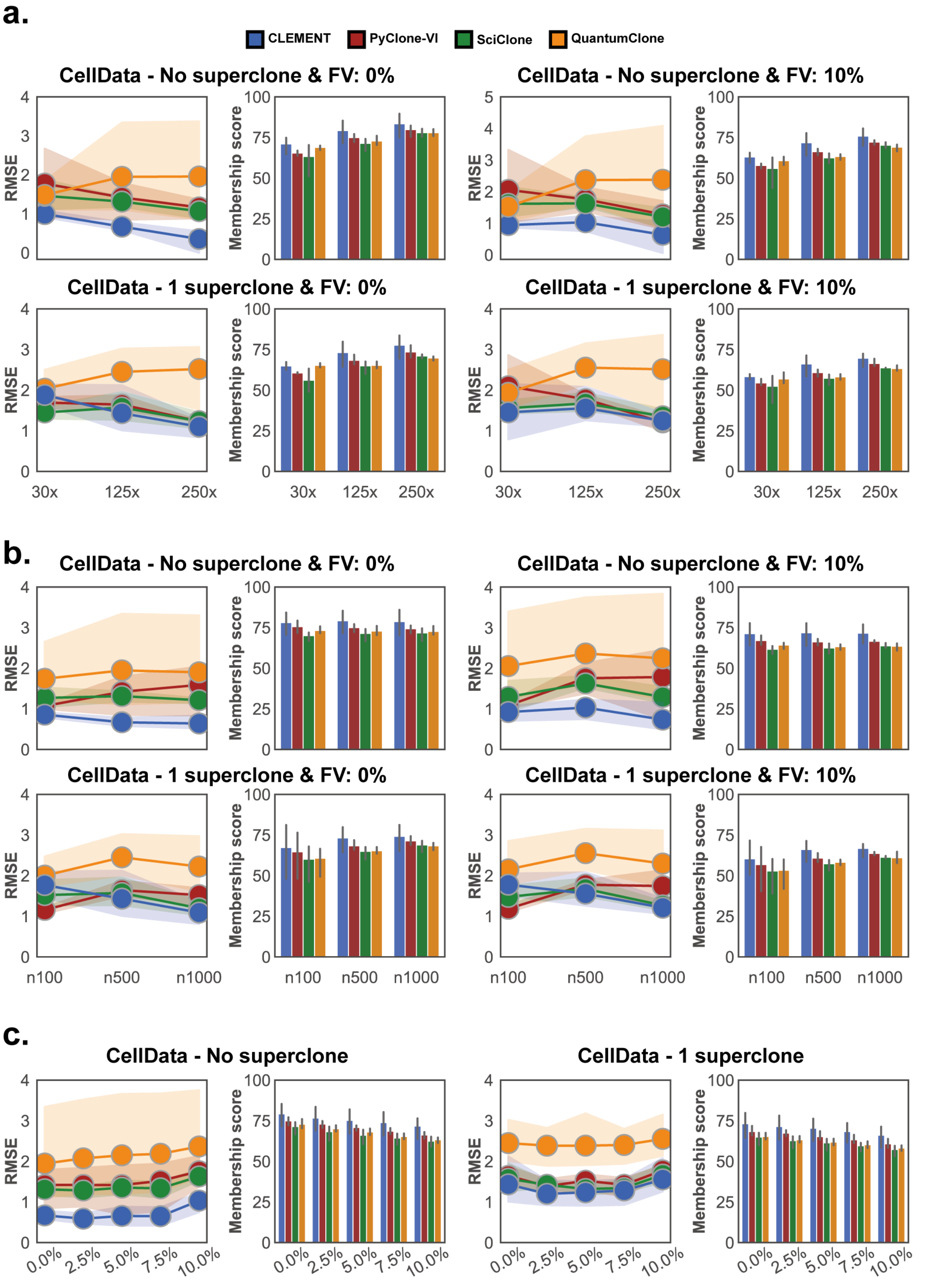

Figure S9. (BioData) Clonal decomposition for bi- or poly-clonal samples of human microdissected normal tissues.

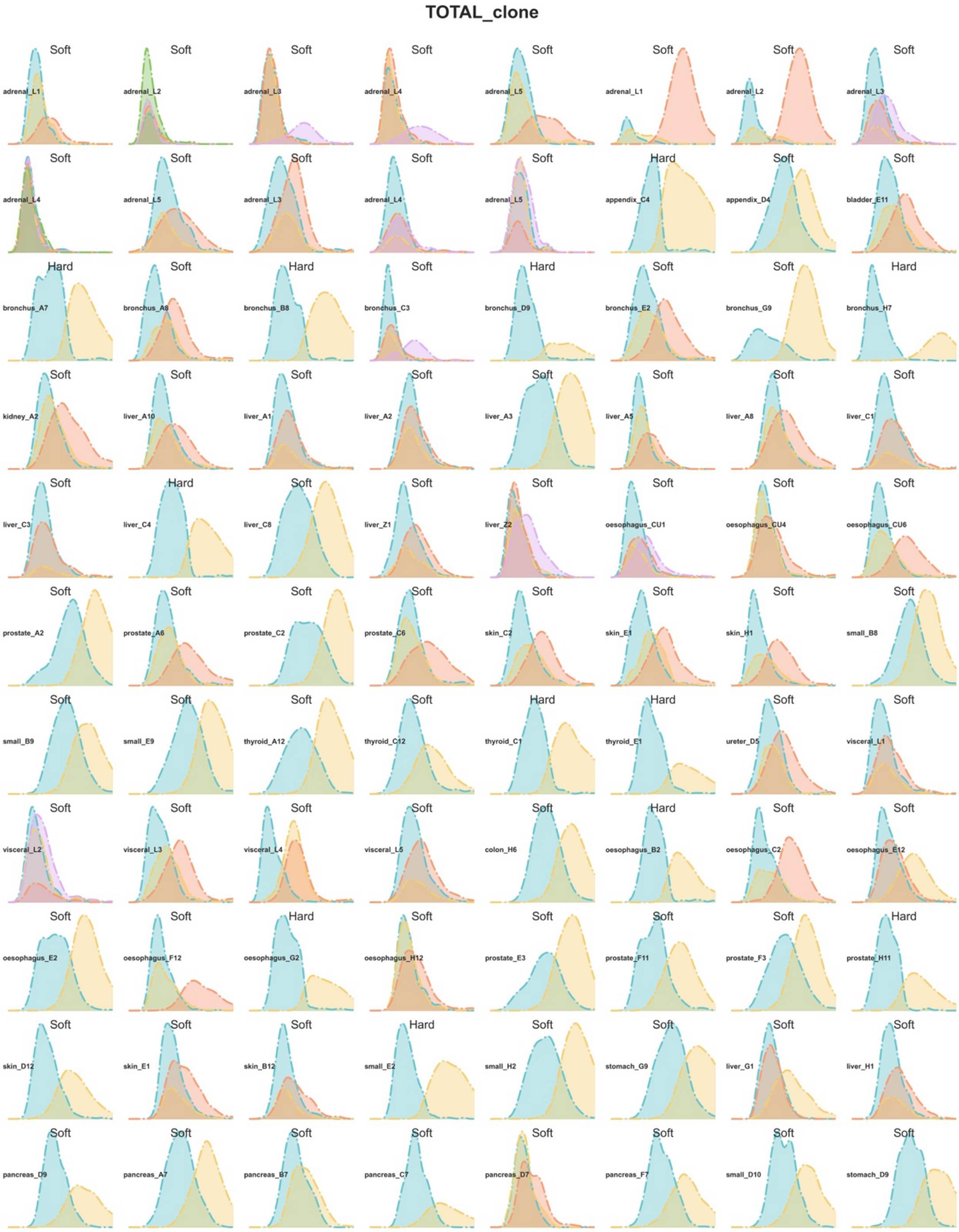

Figure S10. (BioData) Jaccard similarity between L1–L5 locations within each zone.

(a) Zona Glomerulosa (ZG), (b) Zona Fasciculata (ZF), and (c) Zona Reticularis (ZR)

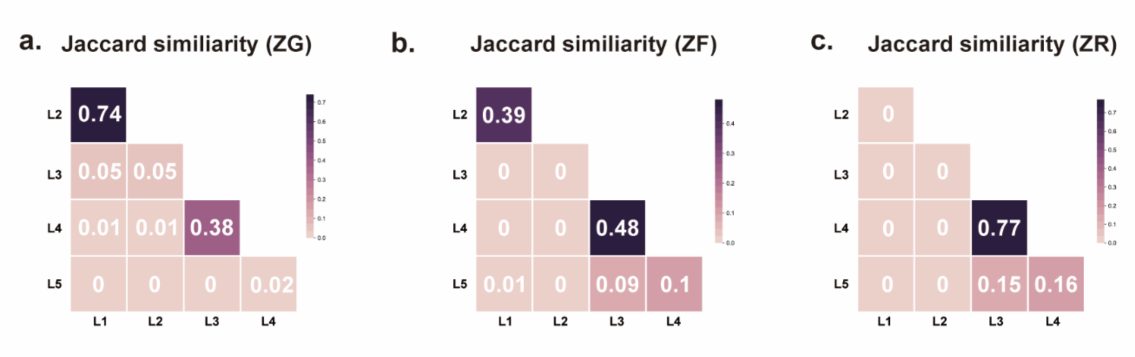

Figure S11. (BioData) One-sample decomposition by CLEMENT in adrenal gland and shared mutations between ZG and ZF in five locations (L1–L5).

ZG: Zona Glomerulosa, ZF: Zona Fasciculata

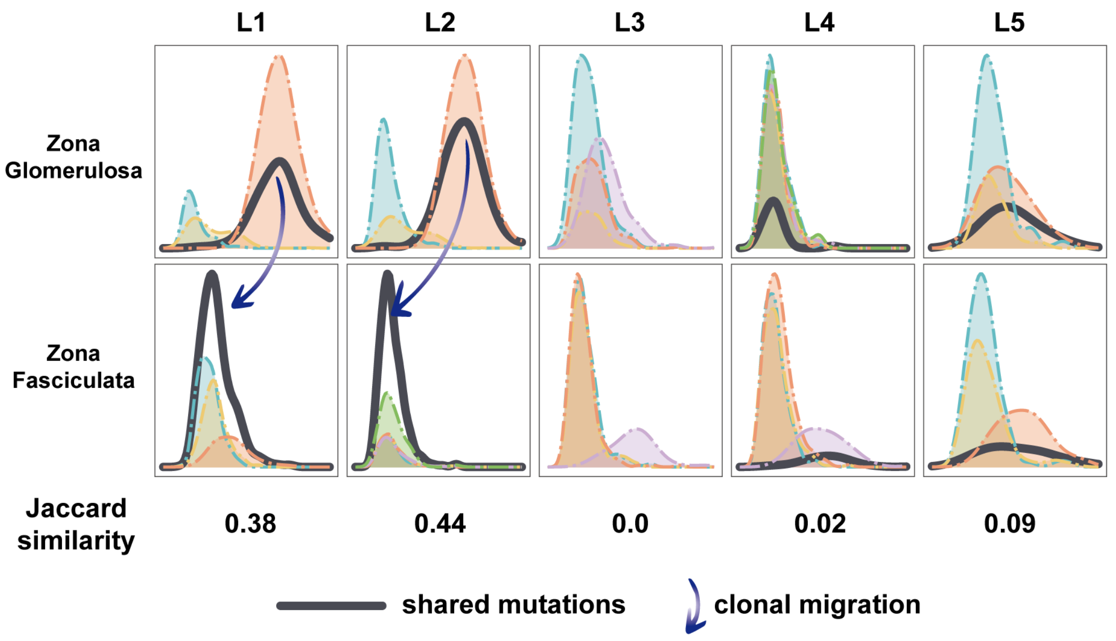

Figure S12. (BioData) Basic statistics for 15 adrenal gland tissues.

(a) Total variant count, (b) median variant allele frequency (VAF), (c) median read-depth, and (d) median alternate allele counts

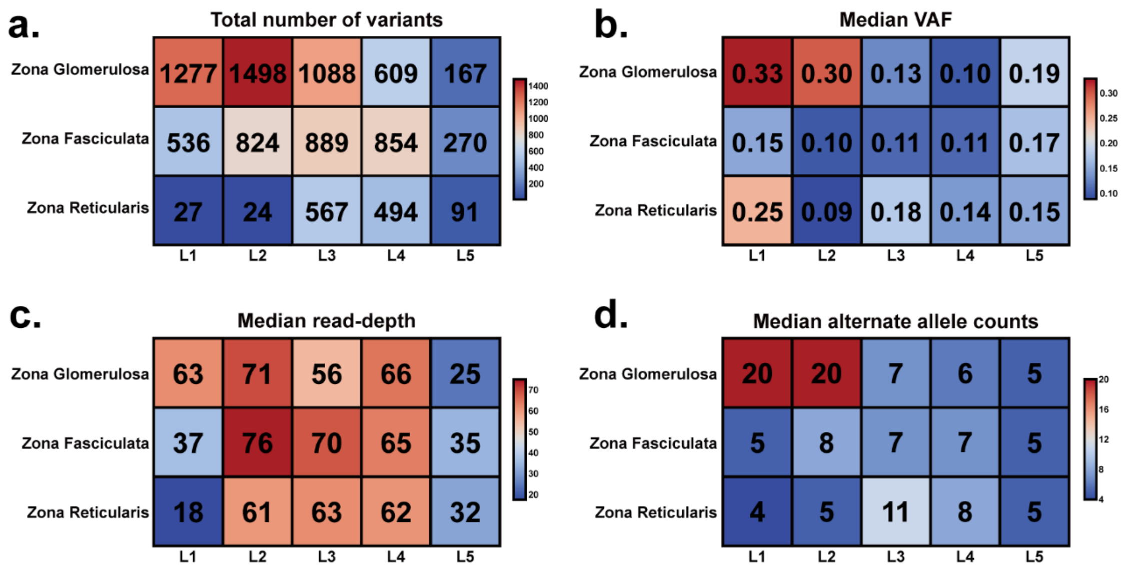

Figure S13. (Discussion) Convergence of CLEMENT by iteration.

(a) In hard clustering, (b) In fuzzy (soft) clustering

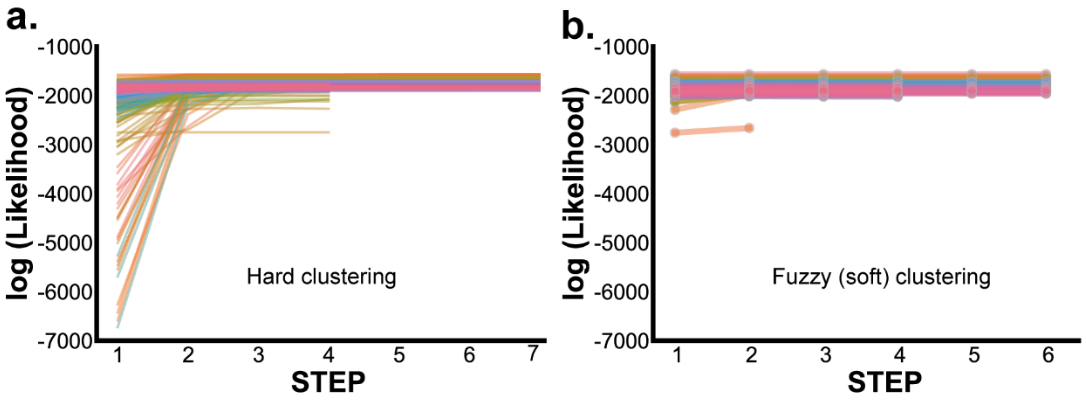

Supplementary Tables

Table S1. (Method) Characteristics of CellData (clonal proportions and number of total variants).

FV: False Variants

		Clones				
	**Proportion(%)**	**S0**	**V1**	**V2**	**V3**	**V4**
**Mixture**	**M1-2**	86%	8%	6%		
	**M1-4**	78.8%	19.2%	2%		
	**M1-6**	30.8%	19.2%	50%		
	**M1-8**	38%	56%	6%		
	**M2-2**	76%	8%		10%	6%
	**M2-4**	70%	8%		16%	6%
	**M2-6**	64.8%	19.2%		10%	6%
	**M2-8**	58.8%	19.2%		16%	6%

		Clones						
	**Number of mutations**	**S0**	**V1**	**V2**	**V3**	**V4**	**Ancestral clone**	**FV**
**Mixture**	**M1-2**	1150	1540	2347			113	196
	**M1-4**	1156	2557	423			120	175
	**M1-6**	1011	2160	6404			115	239
	**M1-8**	1072	2727	4196			121	206
	**M2-2**	1151	1634		1533	875	317	211
	**M2-4**	1150	1651		1913	1068	320	227
	**M2-6**	1140	2608		1652	1145	322	196
	**M2-8**	1142	2570		1930	973	328	200

Table S2. (SimData) Demonstration of the superiority of CLEMENT over other cancer decomposition tools (PyClone-VI, SciClone, and QuantumClone) in terms of the Adjusted Rand Index (ARI) in various situations.

SimData with 500 mutations, 125 read-depth and no false variant embedded; ARI is in-applicable in the presence of false variants.

	One-sample	Two-sample	Three-sample
**Decoy**	0.55 vs. 0.51-52	0.83 vs. 0.74-76	0.93 vs. 0.84-90
**Lump**	0.39 vs. 0.34-37	0.66 vs. 0.53-57	0.79 vs. 0.66-70

Table S3. (SimData) Detection rate of false variants in various situations.

Definition of “detection” is established when CLEMENT isolated more than 20 false variants in datasets with 500 mutations, 125 read-depths and 50 false variants embedded.

	One-sample	Two-sample	Three-sample
**Decoy**	~2.1%	~33.3%	~87.2%
**Lump**	~14.6%	~60.4%	~97.9%

Table S4. (CellData) Demonstration of the superiority of CLEMENT over other cancer decomposition tools (PyClone-VI, SciClone, and QuantumClone) in terms of the Adjusted Rand Index (ARI) in various situations.

CellData with 500 mutations, 125 read-depths and no false variant embedded; ARI is inapplicable in the presence of false variants.

	One-sample	Two-sample	Three-sample
**No ancestral clone**	0.49 vs. 0.46-52	0.7 vs. 0.61-64	0.8 vs. 0.62-70
**1 ancestral clone**	0.42 vs. 0.43-48	0.65 vs. 0.47-62	0.74 vs. 0.59-68

Table S5. (CellData) Detection rate of false variants in CellData.

Definition of “detection” is established when CLEMENT isolated more than 20 false variants in datasets with 500 mutations, 125 read-depths and 50 false variants embedded.

	One-sample	Two-sample	Three-sample
**No ancestral clone**	unavailable	~29.5%	~45.1%
**1 ancestral clone**		~18.8%	~49.1%
